# Impact of preoperative anemia on patients undergoing total joint replacement of lower extremity: a systematic review and meta-analysis

**DOI:** 10.1186/s13018-024-04706-y

**Published:** 2024-04-18

**Authors:** Fu-Qiang Zhang, Yong-Ze Yang, Peng-Fei Li, Guo-Rong Ma, An-Ren Zhang, Hui Zhang, Hong-Zhang Guo

**Affiliations:** 1 First Clinical Medical College of Gansu, University of Traditional Chinese Medicine, Lanzhou, China; 2 People’s Hospital of Gansu Province, Chengguan District, 204 Donggang West Road, Lanzhou, 730000 China

**Keywords:** Anemia, Arthroplasty, Replacement, Knee, Arthroplasty, Replacement, Hip, Treatment outcome, Meta-analysis, Preface

## Abstract

**Purpose:**

Preoperative anemia increases postoperative morbidity, mortality, and the risk of allogeneic transfusion. However, the incidence of preoperative anemia in patients undergoing total hip arthroplasty and total knee arthroplasty (TKA) and its relationship to postoperative outcomes has not been previously reported.

**Methods:**

We conducted a comprehensive literature search through PubMed, Cochrane Library, Web of Sincien, and Embase from inception to July 2023 to investigate the prevalence of preoperative anemia in patients undergoing Total Joint Arthroplasty, comorbidities between anemic and non-anemicpatients before surgery, and postoperative outcomes. postoperative outcomes were analyzed. Overall prevalence was calculated using a random-effects model, and heterogeneity between studies was examined by Cochran's Q test and quantified by the *I*^*2*^ statistic. Subgroup analyses and meta-regression analyses were performed to identify sources of heterogeneity. Publication bias was assessed by funnel plots and validated by Egger's test.

**Results:**

A total of 21 studies with 369,101 samples were included, all of which were retrospective cohort studies. 3 studies were of high quality and 18 studies were of moderate quality. The results showed that the prevalence of preoperative anemia was 22% in patients awaiting arthroplasty; subgroup analyses revealed that the prevalence of preoperative anemia was highest in patients awaiting revision of total knee arthroplasty; the highest prevalence of preoperative anemia was found in the Americas; preoperative anemia was more prevalent in the female than in the male population; and preoperative anemia with a history of preoperative anemia was more common in the female than in the male population. patients with a history of preoperative anemia; patients with joint replacement who had a history of preoperative anemia had an increased risk of infection, postoperative blood transfusion rate, postoperative blood transfusion, Deep vein thrombosis of the lower limbs, days in hospital, readmission within three months, and mortality compared with patients who did not have preoperative anemia.

**Conclusion:**

The prevalence of preoperative anemia in patients awaiting total joint arthroplasty is 22%, and is higher in TKA and female patients undergoing revision, while preoperative anemia is detrimental to the patient's postoperative recovery and will increase the risk of postoperative complications, transfusion rates, days in the hospital, readmission rates, and mortality.

**Supplementary Information:**

The online version contains supplementary material available at 10.1186/s13018-024-04706-y.

## Preface

The ability to recover after arthroplasty depends on the patient’s preoperative status. Preoperative hemoglobin is a functional reserve component that can be altered [1–3]. At the same time, preoperative anemia is relatively common in patients undergoing elective arthroplasty (15–30%) [4] and is associated with a poor prognosis after primary arthroplasty and revision. The World Health Organization (WHO) defines anemia as a hemoglobin (HB) concentration < 120 g/L in non-pregnant women and < 130 g/L *in* men.Untreated preoperative anemia during surgery is associated with increased postoperative complications, mortality, length of hospital stay, and a threefold increase in the risk of requiring allogeneic blood transfusion (ABT) [5–8]. Allogeneic blood transfusion is associated with inherent risks including infection, delayed wound healing, fluid overload, and transfusion-associated lung injury (TRALI) [9]. The risk of allogeneic blood transfusion is associated with inherent risks including infection, delayed wound healing, fluid overload, and transfusion-associated lung injury (TRALI). ABT is also associated with prolonged hospitalization [10, 11], and blood products are expensive[12]. Therefore, it is important to understand and address preoperative anemia from clinical and health economics perspectives.

Currently, total lower extremity arthroplasty, including THA, TKA, revision of total hip arthroplasty (rTHA), and revision of total knee arthroplasty (rTKA), is a very popular and safe procedure for the treatment of osteoarthritis. With significant advances in surgical techniques and implant design, coupled with an increasingly aging population, the demand for lower-extremity arthroplasty continues to increase [13]. However, anemia is prevalent in older patients undergoing TJA, one study reported that 44% of patients admitted to the hospital awaiting total joint arthroplasty were anemic, with this percentage increasing to 87% postoperatively [4]. In addition, the aging population means that more patients with increasing frailty and comorbidities, such as anemia, are requiring hip and knee replacement. Increased complications and mortality after primary and revision TJA are associated with preoperative anemi [14]. Patients with severe preoperative anemia before TKA are at significant risk of postoperative DVT, sepsis, wound infection, and wound stemming [15]. Preoperative anemia has also been shown to be a risk factor for increased economic burden after TJA owing to higher transfusion rates, longer hospital stays, and transfusion-related complications [16, 17]. Patients with moderate to severe anemia are more likely to have postoperative complications than those with mild anemia, and there is a significant correlation between increased postoperative complications and the severity of anemia in patients undergoing TJA [17]. Therefore, we conducted this systematic review and meta-analysis, which is the first study to summarize the incidence of preoperative anemia and postoperative clinical outcomes in patients undergoing primary or revision total knee and hip arthroplasty.

The main aim of this systematic review and meta-analysis was to investigate the prevalence of preoperative anemia in patients awaiting total lower limb arthroplasty, and the impact of preoperative anemia on clinical outcomes following total joint arthroplasty. This study extends our understanding of the relationship between preoperative anemia and subsequent arthroplasty. We hypothesized that patients with preoperative anemia would have similar outcomes after THA or TKA compared with patients without preoperative anemia.

## Materials and methods

The published literature was comprehensively reviewed and reported by the Assessing Methodological Quality in Systematic Reviews (AMSTAR) [18] and Preferred Reporting Items for Systematic Reviews and Meta-Analyses (PRISMA) guidelines, which include requirements essential for transparent reporting of results [19]. Our research protocol was registered in (Prospero: CRD42023443351) for literature selection, eligibility criteria evaluation, data extraction, and analysis.

### Search strategy

We conducted a literature search in PubMed, Web of Science, Cochrane Library, and EMbass to analyze the prevalence of preoperative anemia in patients undergoing total hip replacement or total knee arthroplasty from inception to July 2023 and the impact on clinical outcomes. Keywords and Medical Subject Headings (MeSH) terms were used in the search, and the following search terms were used in various combinations: “anemia”, “preoperative”, “total knee arthroplasty”, “total hip arthroplasty”, “TKA”, and “THA”. To broaden the scope of the retrieval, no restrictions were set for the language, and relevant articles were found as comprehensively as possible. The search strategy for the four databases is detailed in Additional file 1: Appendix 1. Two independent authors performed all searches to identify studies related to THA or TKA. During the full-text review stage, the reviewers discussed discrepancies until a consensus was reached.

### Eligibility criteria

Two authors were independently screened for eligibility to participate in the study based on the title and abstract. Full articles were reviewed based on the inclusion and exclusion criteria. Any disagreements during the selection process were resolved through discussion between the two authors and another professor.

1. The inclusion criteria are as follows:

1. Cross-sectional or longitudinal observational study.
2. at least one finding was reported in this study.
3. The comparisons listed should include patients with preoperative anemic and preoperative non-anemic.
4. Articles identified in any language type.
5. Full-text articles can be accessed.

2. Exclusion criteria such as:

1. Reviews, conference abstracts, case reports, letters to the editor, journal articles, or commentaries.
2. Studies that were unavailable in full or did not provide sufficient data on the prevalence of anemia.
3. It was not possible to extract raw data from the comparison results.

### Data extraction

Before the start of the study, two reviewers independently extracted and recorded the extracted data in a collaborative online spreadsheet (Excel sheet). A third reviewer repeated the data extraction and compared the results for validation. The reviewer recorded the first author, year of publication, study design, sample size, type of surgery, characteristic sample (including age, number of males and females, and MBI), and article type. Outcomes included the prevalence of preoperative anemia and its relation to perioperative blood transfusion, number of postoperative blood transfusions, preoperative comorbidities involving a history of hypertension (HTN), diabetes mellitus (DM), chronic obstructive pulmonary disease (COPD), preoperative comorbidities including DVT, infections (superficial and deep), and in-hospital mortality.T1DM, T2DM, and other secondary DM (e.g., insulin-dependent DM and non-insulin-dependent DM) were not analyzed separately; all subgroups were categorized as DM groups in our study. In studies where meta-analysis data were missing or unavailable, or data were presented only graphically, attempts were made to contact the corresponding authors by email. If necessary, the need for extraction of incomplete data was waived, and when disagreements arose during data collection, they were resolved by discussion.

### Quality assessment

The literature search did not yield randomized studies. The Newcastle–Ottawa Scale (NOS) was used to assess the methodological quality of non-randomized case–control studies [20] which consists of eight items with a total score of 9. “Good” was defined as a total score of 7–9, “Fair” as a score of 4–6, and “Poor” as a score of less than 4. All the selected articles were independently reviewed by two authors for quality assessment. Disagreements were resolved by discussion. Kappa scores for the inter-reviewer agreement were as follows: < 0.2 normal, 0.40–0.59; good, 0.60–0.74; and very good, ≥ 0.75.

### Quality of evidence

The Grading of Recommendations, Assessment, Development, and Evaluation (GRADE) framework was used to assess the quality of evidence for outcomes [21]. Evidence may be reduced by five factors: study limitations, inconsistency, indirectness, imprecision, and publication bias; factors that may improve the quality of evidence from observational studies: large effect sizes, negative bias, and dose–effect relationships. The results of studies with moderate or large effect sizes may lead to an improved quality of evidence. Four quality levels were used: high, moderate, low, and very low quality.

### Statistical analyses

We used R software (4.3) to determine pooled prevalence and performed a meta-analysis of preoperative anemia in patients awaiting hip or knee replacement. Given the high degree of heterogeneity expected in observational studies, we used a random-effects model to calculate the pooled estimates. To stabilize the variance, the study data were transformed using the Freeman-Tukey double-orthogonal string transformation. We analyzed heterogeneity using Cochran’s Q test, and the *I*^*2*^ statistic with a threshold of *I*^2^ ≥ 50% heterogeneity was considered high. We conducted a subgroup analysis of the factors that may influence the prevalence of preoperative anemia in total joint arthroplasty to explore the sources of heterogeneity, namely gender, type of surgery, and continent; this is due to differences in gender, as females have lower hemoglobin levels than males, and changes in the physiological cycle of females make females more susceptible to anemia; and the causes of preoperative anemia are more complex in revision joints compared with first-time total joint arthroplasty. The causes of preoperative anemia in revision arthroplasty are more complex, and may be related to the combination of long-term chronic infection and inflammation; in addition, the incidence of anemia varies in different regions due to the differences in economic level, medical level, the standard of living, and dietary habits of different regions, and therefore, the incidence of anemia is different in different regions according to gender, type of surgery (THA, TKA, rTHA, rTKA), and continent (Europe, Asia, and America), Asia, America) on anemia was analyzed in subgroups. When meta-analyzing preoperative anemia subgroups, preoperative comorbidities, and postoperative clinical outcomes, dichotomous outcomes or continuous outcomes were assessed using relative risk (RR) with 95% confidence intervals (CI) or standardized mean differences (MD) with 95% confidence regions, respectively. A significance level of *P* < 0.05 was used, and *I*^2^ was used to evaluate heterogeneity. If *I*^2^ < 50%, a fixed-effects model was used because of low heterogeneity. *I*^2^ ≥ 50% was considered significant heterogeneity, and a random-effects model was used to calculate pooled estimates. Sensitivity analyses were performed by sequentially deleting studies to determine the source of heterogeneity [22]. Publication bias was assessed by visual inspection of funnel plots and Egger’s test [23].

## Results

### Literature search and characteristics

**Fig. 1:**
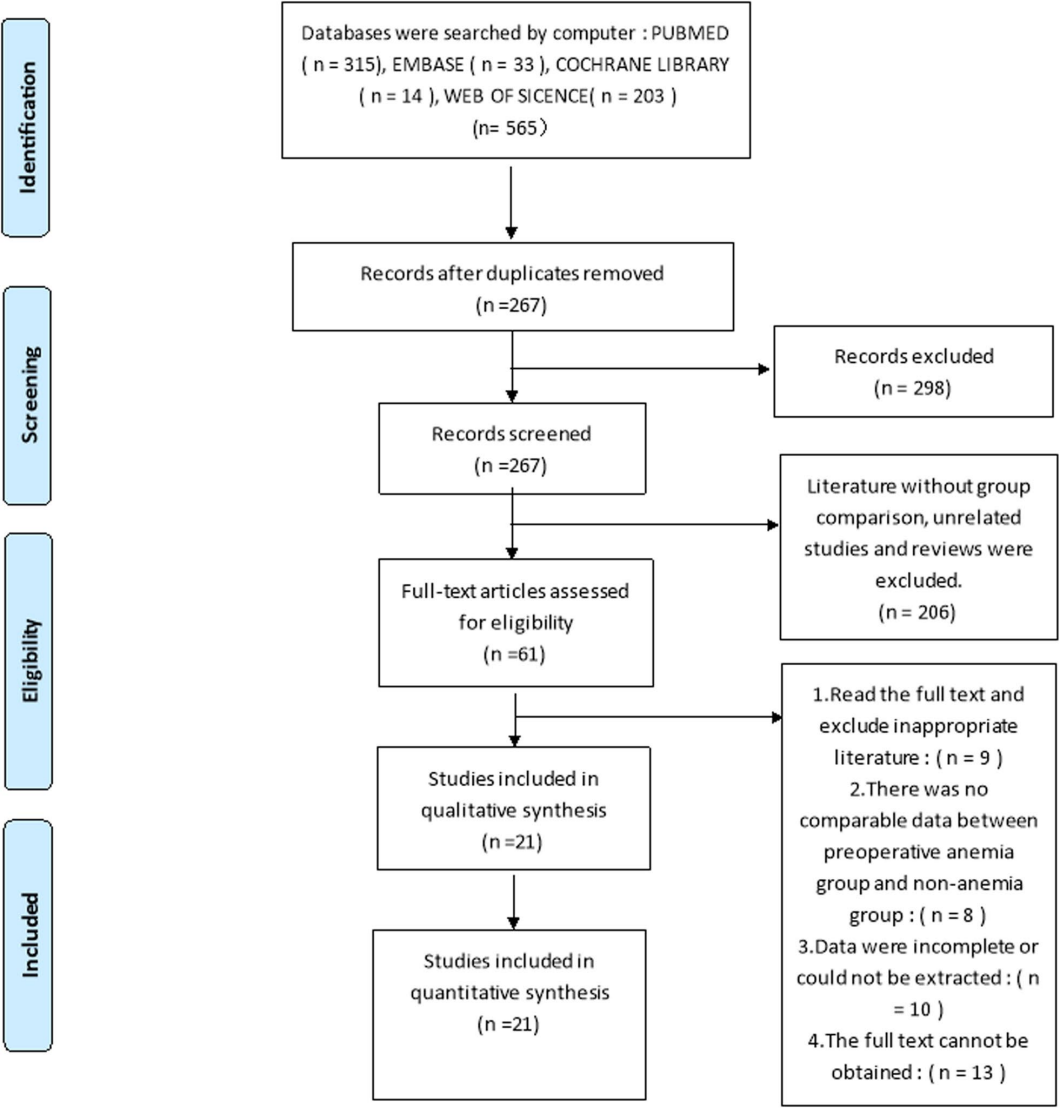
Preferred reporting items of systematic reviews and met-analysis (PRISMA) flow diagram

Figure 1 summarizes the search and selection process. An electronic search yielded 565 citations from the database. A total of 298 citations were removed because of duplication, and 216 citations were excluded by title and abstract screening. A total of 51 articles were selected for full-text screening. Nine were read in full text to exclude inappropriate literature, 8 had no comparable information between the preoperative anemic and non-anemic groups, 10 had incomplete or unavailable data, and 13 were unavailable in full text. Finally, this study ultimately included 21 studies published between 2003 and 2019 studies were included in the qualitative and quantitative synthesis.The total number of patients included in this meta-analysis was 369,101, of whom 162,480 were women and 206,621 were men. The number of patients in each study ranged from 154 to 293,043. Studies were conducted on four continents and in 10 countries: the United Kingdom [24–29], China [30], Denmark [1], United States [14, 17, 31, 32], Singapore [7, 33], France [34], Australia [35], Germany [36, 37], Canada [38] and Brazil [39]. The mean age range for inclusion in the studies was 63.1 ± 11.7 years to 74.06 ± 1.5 years. A total of 369,101 patients who underwent total joint arthroplasty were enrolled, including 56,175 patients with preoperative anemia and 312,926 patients without preoperative anemia. 21 Twenty-one studies had confirmed articles describing patients treated with primary THA or TKA. Specifically, nine studies [1, 17, 26, 29, 31, 32, 34, 38, 40] investigated TKA, 9 studies [1, 17, 28, 30–32, 34, 38, 40] investigated THA, and 5 studies [14, 24, 31, 32, 40] investigated rTKA and rTHA. Detailed characteristics of each study are presented in Table 1.

**Table 1:** Characteristics of the included studies

Author(s) (etc.) Year	Nations	Type of study	research period	Type of surgery	sample size	Number of men/women	Age (mean (SD); Range (years)	BMI (kg/m^2^)	Anaemia criteria	Number of preoperative anemia	Outcome
Sandean et al. [28]	United Kingdom	A retrospective study	2009–2017	THA	THA:1095	unspecified	72	unspecified	< 130 g/L for men or < 115 g/L for women	192	1
Xiong et al. [30]	China	A retrospective study	2017–2021	TKA	TKA:1005	208/797	Anaemic group: 69.87 ± 8.60 Non-anaemic group: 69.87 ± 8.60	Anaemia group: 24.87 ± 3.57 Non-anaemic group 25.81 ± 2.46	Hb < 130 g/L for men or < 120 g/L for women	342	1, 2, 8, 9
Sandean et al. [29]	United Kingdom	A retrospective study	2016–2018	TKA	TKA:2296	883/1413	72	unspecified	< 130 g/L for men or < 115 g/L for women	350	1
Saleh et al. [27]	United Kingdom	A retrospective study	2000–2001	THA/TKA	THA:621 TKA:521	unspecified	68	unspecified	< 130 g/L for men or < 115 g/L for women	210	1
Lasocki et al. [34]	French	A retrospective study	2010–2011	THA/TKA	THA:765 TKA:570	397/938	64 ± 12.3	unspecified	Hb < 130 g/L for men and < 120 g/L for women	217	1, 7, 10
Myers et al. [25]	United Kingdom	A retrospective study	1999–2000	THA	THA:225	137/88	Anaemic group: 62 Non-anaemic group: 64	unspecified	< 130 g/L for men or < 115 g/L for women	35	1, 2, 13
Jans et al. [1]	Denmark	A retrospective study	2010–2011	THA/TKA	THA:2702 TKA:2463	2229/2936	67 ± 11	unspecified	Hb < 130 g/L for men and < 120 g/L for women	662	1, 2, 3, 4, 7, 8, 10, 11
Lu et al. [14]	United States of America	A retrospective study	2006–2014	rTHA/rTKA	rTHA:3871 rTKA:2959	3814/3016	Anaemia group: 68.1 ± 12.1 Non-anaemic group:67.9 ± 11.4	Anaemia group: 31 ± 7 Non-anaemic group: 30.9 ± 8	Hb < 130 g/L for men and < 120 g/L for women	3415	1, 2, 3, 4, 5, 6, 8, 9, 10, 11
Lu et al. [14]	United States of America	A retrospective study	2006–2014	rTHA/rTKA	rTHA:1107 rTKA:1543	1364/1286	Anaemia group: 64.3 ± 11.7 Non-anaemic group:66.3 ± 12	Anaemia group: 31.9 ± 7.6 Non-anaemic group 31.6 ± 8	Hb < 130 g/L for men and < 120 g/L for women	1325	1, 2, 3, 4, 5, 6, 8, 9, 10, 11
Kasivisvanathan et al. [24]	United Kingdom	A retrospective study	2004–2014	rTHA/rTKA	rTHA:3021 rTKA:2366	2409/2978	70.1 ± 8.4	unspecified	Hb < 130 g/L for men and < 120 g/L for women	1956	1, 2, 3, 4, 8, 10, 12
Abdullah et al. [7]	Singaporean	A retrospective study	2013–2014	TKA	TKA:2394	579/1815	65.9 ± 8.0	unspecified	Hb < 130 g/L for men and < 120 g/L for women	567	1
Evans et al. [35]	Australia	A retrospective study	2009.1–2009.7	TJA	TJA:154	69/85	66.35	unspecified	Hb < 130 g/L for men and < 120 g/L for women	15	1, 8, 13
Wan et al. [40]	Sweden	A retrospective study	2016–2018	THA/TKA/ rTHA/rTKA	881	533/348	Anaemia group: 69.5 Non-anaemic group: 65.2	unspecified	Hb < 130 g/L for men and < 120 g/L for women	189	1, 5, 8, 10, 12
Gu et al. [17]	United States of America	A retrospective study	2012–2017	THA	THA:108 b,966	48,743/60223	Anaemic group: 64.63 ± 10.715 Non-anaemic group: 68.01 ± 11.49	Anaemia group: 29.9 ± 6.53 Non-anaemic group 30.5 ± 6.35	Hb < 130 g/L for men and < 120 g/L for women	14,751	1, 2, 3, 4, 6, 8, 9, 11, 12
Gu et al. [17]	United States of America	A retrospective study	2012–2017	TKA	TKA:184,077	69,415/114662	Anaemic group: 66.34 ± 9.34 Non-anaemic group: 68.80 ± 9.84	Anaemia group: 32.6 ± 7.16 Non-anaemic group 33.2 ± 6.84	Hb < 130 g/L for men and < 120 g/L for women	23,637	1, 2, 3, 4, 6, 8, 9, 11, 12
Greenky [31]	United States of America	A retrospective study	2000–2007	THA/TKA/ rTHA/rTKA	THA: 7230 TKA: 6371 rTHA: 1121 rTKA: 500	6494/8727	Anaemic group: 65.92 ± 12.9 Non-anaemic group: 63.14 ± 12.2	Anaemic group: 29.7 ± 8.32 Non-anaemic group 30.27 ± 10.7	Hb < 130 g/L for men and < 120 g/L for women	2991	1, 2, 3, 4, 5, 9, 10, 12
Rogers et al. [26]	United Kingdom	A retrospective study	unspecified	THA	THA:322	141/181	67	unspecified	Male and female Hb ≤ 120 g/L	26	1
Duarte et al. [39]	Brazilian	A retrospective study	2017–2020	TJA	TJA:234	99/134	Anaemic group: 74.06 ± 1.59 Non-anaemic group: 68.84 ± 1.29	unspecified	Hb < 130 g/L for men and < 120 g/L for women	72	1, 7, 8, 10, 11, 12
Schatz et al. [37]	German	A retrospective study	2019–2020	TKA	TKA:341	147/126	71.3 ± 9.0	Anaemia group: 26.7 ± 4.6 Non-anaemic group 28.4 ± 4.6	Hb < 130 g/L for men and < 120 g/L for women	42	1,
Abdullah et al. [33]	Singaporean	A retrospective study	2013–2014	TKA	TKA:1994	473/1521	67.3	unspecified	Hb < 130 g/L for men and < 120 g/L for women	445	1, 3, 8,
Bailey et al. [38]	Canadian	A retrospective study	2016–2017	THA/TKA	THA:2283 TKA:2457	2417/2967	Anaemic group: 70.4 ± 11.4 Non-anaemic group: 63.7 ± 11.7	Anaemia group: 30.6 ± 6.7 Non-anaemic group 31.1 ± 7.0	Hb < 130 g/L for men and < 120 g/L for women	817	1, 5, 6, 8, 11
Viola et al. [32]	United States of America	A retrospective study	2000–2013	THA/TKA/ rTHA/rTKA	THA: 6320 TKA: 5619 rTHA: 1012 rTKA: 612	5962/7601	Anaemia group: 66.1 ± 12.4 Non-anaemic group: 63.1 ± 11.7	Anaemic group: 29.9 ± 6.9 Non-anaemic group: 30.3 ± 6.4	Hb < 130 g/L for men and < 120 g/L for women	2576	1, 8, 10, 12
Meybohm et al. [36]	German	A retrospective study	2017–2018	THA/TKA	THA:4813 TKA:3162	3382/4593	70	unspecified	Hb < 130 g/L for men and < 120 g/L for women	1372	1, 10, 12

1, prevalence of preoperative anemia in total joint arthroplasty; 2, preoperative combined hypertension; 3, preoperative combined diabetes mellitus; 4, preoperative combined chronic obstructive pulmonary disease; 5, postoperative deep infections; 6, postoperative superficial infections; 7, blood transfusion; 8, transfusion rate; 9, deep vein thrombosis in the lower limbs; 10, number of days in hospital; 11, rehospitalisation rate in 3 months; 12, mortality rate, 13 causes of anaemia

*TJA* *total joint arthroplasty*, *THA* total hip arthroplasty, *TKA*, total knee arthroplasty, *rTHA *revision of total hip arthroplasty, *rTKA* revision of total knee arthroplasty, *IFR* infection-free refurbishment, *IR* infection refurbishment

### Methodological quality

The overall Kappa score for the consistency of methodological quality assessment between the two evaluators was 0.905 (Additional file 3: Annex 3). The quality scores ranged from 4 to 7 (maximum: 9), with a mean score of 5.6. There were 18 'fair' studies [1, 7, 14, 25, 26, 31, 33–36, 38, 39], and 3 ‘good’ studies [17, 24, 32]. The NOS scores for methodological quality for each study are presented in Table 2.

**Table 2:** Quality evaluation results of non-randomised controlled studies

Inclusion in the study	Selection of research subjects				Comparability	Outcome measurement			Rating	GRADE
	1	2	3	4		A	B	C		
Sandean et al. [28]	–	*	*	–	*	*	–	–	4	Fair
Xiong et al. [30]	*	*	*	–	*	*	–	*	5	Fair
Sandean et al. [29]	*	*	*	–	*	*	–	*	6	Fair
Saleh et al. [27]	*	*	*	–	*	*	–	–	5	Fair
Lasocki et al. [34]	*	*	*	–	*	*	–	*	6	Fair
Myers et al. [25]	*	*	*	–	*	*	–	*	6	Fair
Jans et al. [1]	*	*	*	–	*	*	–	*	5	Fair
Lu et al. [14]	–		*	–	*	*	*	*	5	Fair
Kasivisvanathan et al. [24]	*	*	*	–	*	*	*	*	7	Good
Abdullah et al. [7]	*	*	*	–	*	*	–	–	5	Fair
Evans et al. [35]	*	*	*	–	*	*	–	–	5	Fair
Wan et al. [40]	*		*	–	*	*	–	*	4	Fair
Gu et al. [17]	*	*	*	–	*	*	*	*	7	Good
Greenky [31]	*	*	*	–	*	*	–	–	6	Fair
Rogers et al. [26]	*	*	*	–	*	*	–	–	5	Fair
Duarte et al. [39]	*	*	*	–	*	*	–	*	6	Fair
Schatz et al. [37]	*	*	*	–	**	*	–	*	6	Fair
Abdullah et al. [33]	*	*	*	–	*	*	–	–	5	Fair
Bailey et al. [38]	*	*	*	–	*	*	–	*	6	Fair
Viola et al. [32]	*	*	*	–	*	*	*	*	7	Good
Meybohm et al. [36]	*	*	*	–	*	*	–	*	6	Fair

1. Representativeness of the exposure cohort; 2. Selection of unexposed; 3. Determination of exposure; 4. Outcomes not present at the start; A. Outcome B. Adequate follow-up time; C. Adequacy of follow-up

1. Prevalence of anemia before waiting for total joint arthroplasty

**Fig. 2:**
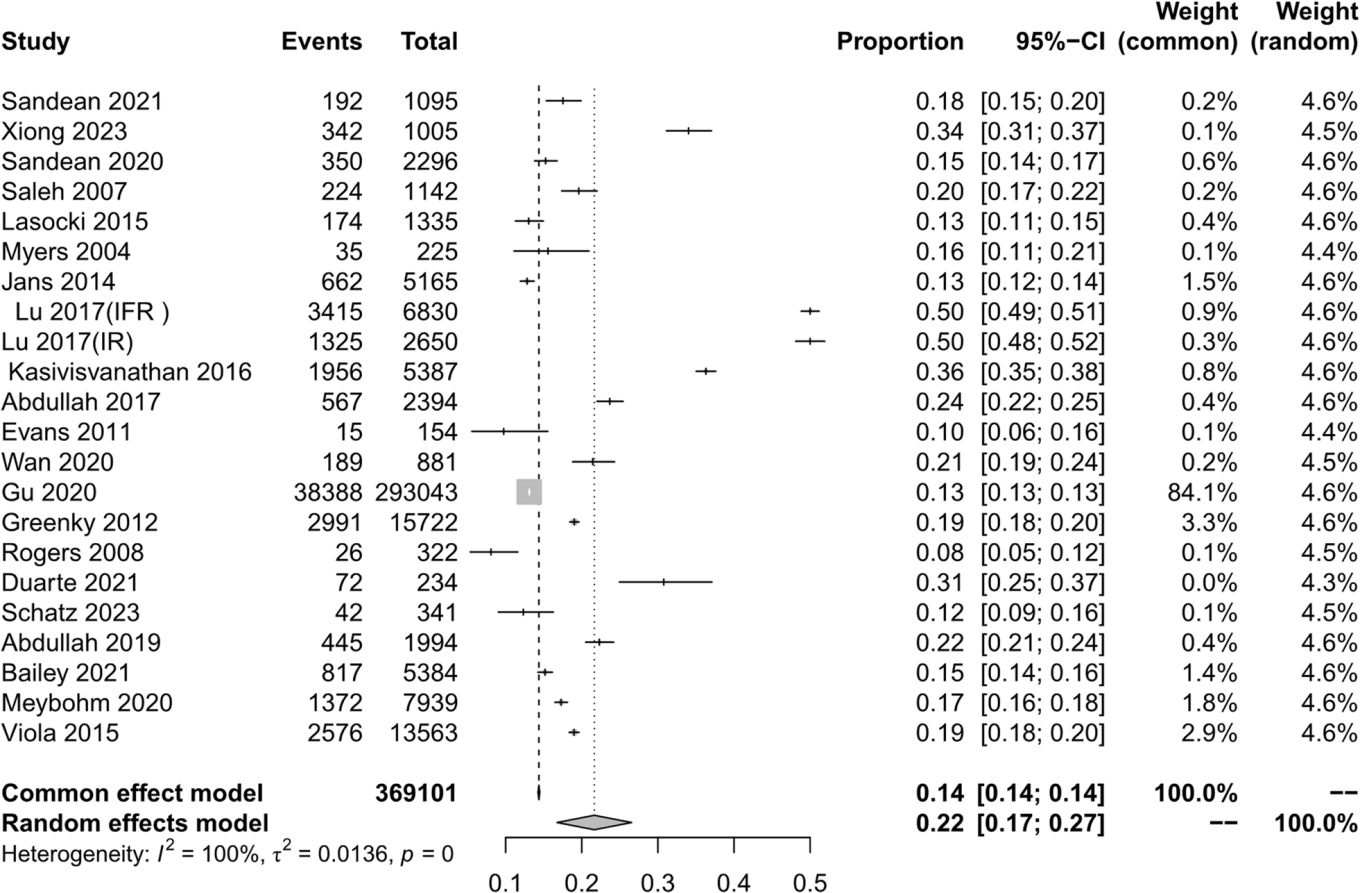
Prevalence of preoperative anaemia in total joint replacement patients

In 21 studies, with a total of 369,101 individuals, the prevalence of combined preoperative anemia in patients undergoing total joint arthroplasty was 22% (95% CI 17–27%; *I*^*2*^ = 100%; *P* < 0.01) (Fig. 2). To explore the sources of heterogeneity, subgroup analyses were performed according to sex, type of surgery, and continent.

2. Analysis of incidence rates by subgroup

1. Type of surgery

**Fig. 3:**
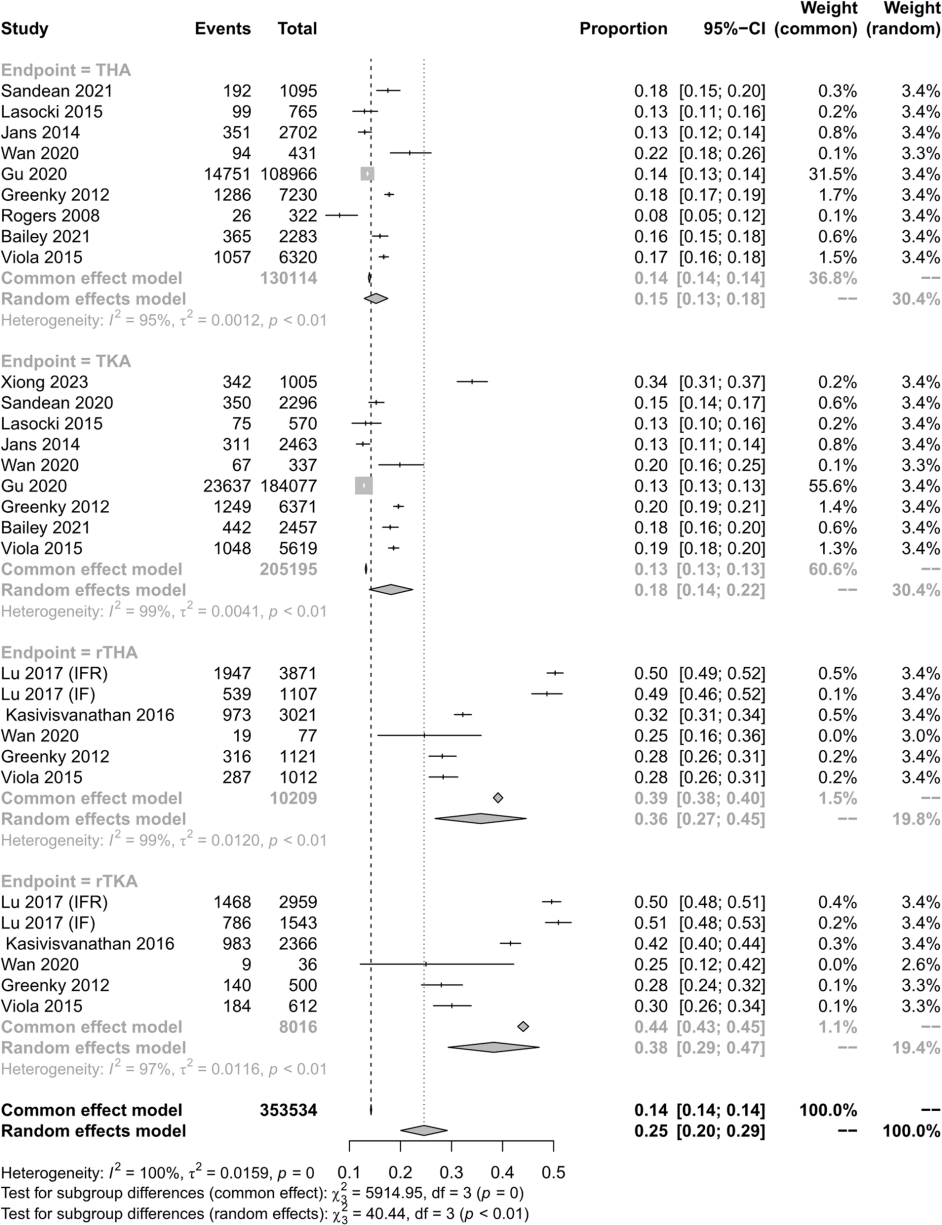
Prevalence of preoperative anaemia for different types of surgery

Nine studies [1, 17, 26, 29, 31, 32, 34, 38, 40] investigated the prevalence of preoperative anemia in THA, nine studies investigated TKA[1, 17, 28, 30–32, 34, 38, 40] investigated the prevalence of preoperative anemia in TKA, five studies[14, 24, 31, 32, 40] investigated the prevalence of preoperative anemia in rTHA, and five studies[14, 24, 31, 32, 40] investigated the prevalence of preoperative anemia in rTKA. The overall prevalence of preoperative anemia in patients with THA, TKA, rTHA, and rTKA combined was 15.2% (95% CI 15.2–17.5%; *I*^2^ = 95 0.3%; *P* < 0.01), 18.2% (95% CI 13.9–22.4%; *I*^2^ = 98.54%; *P* < 0.01), 35.7% (95% CI 13.9–22.4%; *P* < 0.01),* 3*5.7% (95% CI 13.4%; *I*^2^ = 98.54%*; P* < 0.01), 35.7% (95% CI 26.8–44.6%; *I*^2^ = 98.84%; *P* < 0.01) and 38.3% (95% CI 29.3–47.2%; *I*^2^ = 97.5%; *P* < 0.01) (Fig. 3).

(2) Different continents

**Fig. 4:**
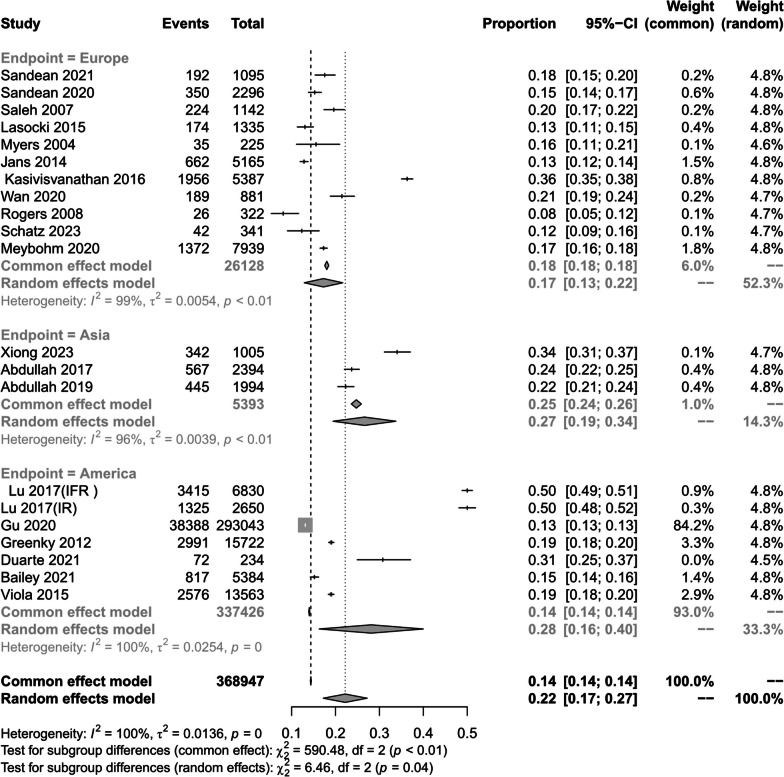
Prevalence of preoperative anaemia in different continents

When we analyzed the prevalence of preoperative anemia according to different continents, significant differences were observed. In Europe [1, 24–29, 34, 36, 37, 40],the prevalence was 16.9%,(95% CI 13.0–21.1%; *I*^2^ = 99.0%; *P* < 0.01); in Asia [7, 30, 33],–the prevalence in Asia was 26.5%, (95% CI 19.7–33.8%; *I*^2^ = 96%; *P* < 0.01); in the Americas, the prevalence was 28.5% [14, 17, 31, 32, 38, 39]. (95% CI 16.1–42.9%; *I*^2^ = 100; *P* = 0.01) (Fig. 4).

(3) Gender differences

**Fig. 5:**
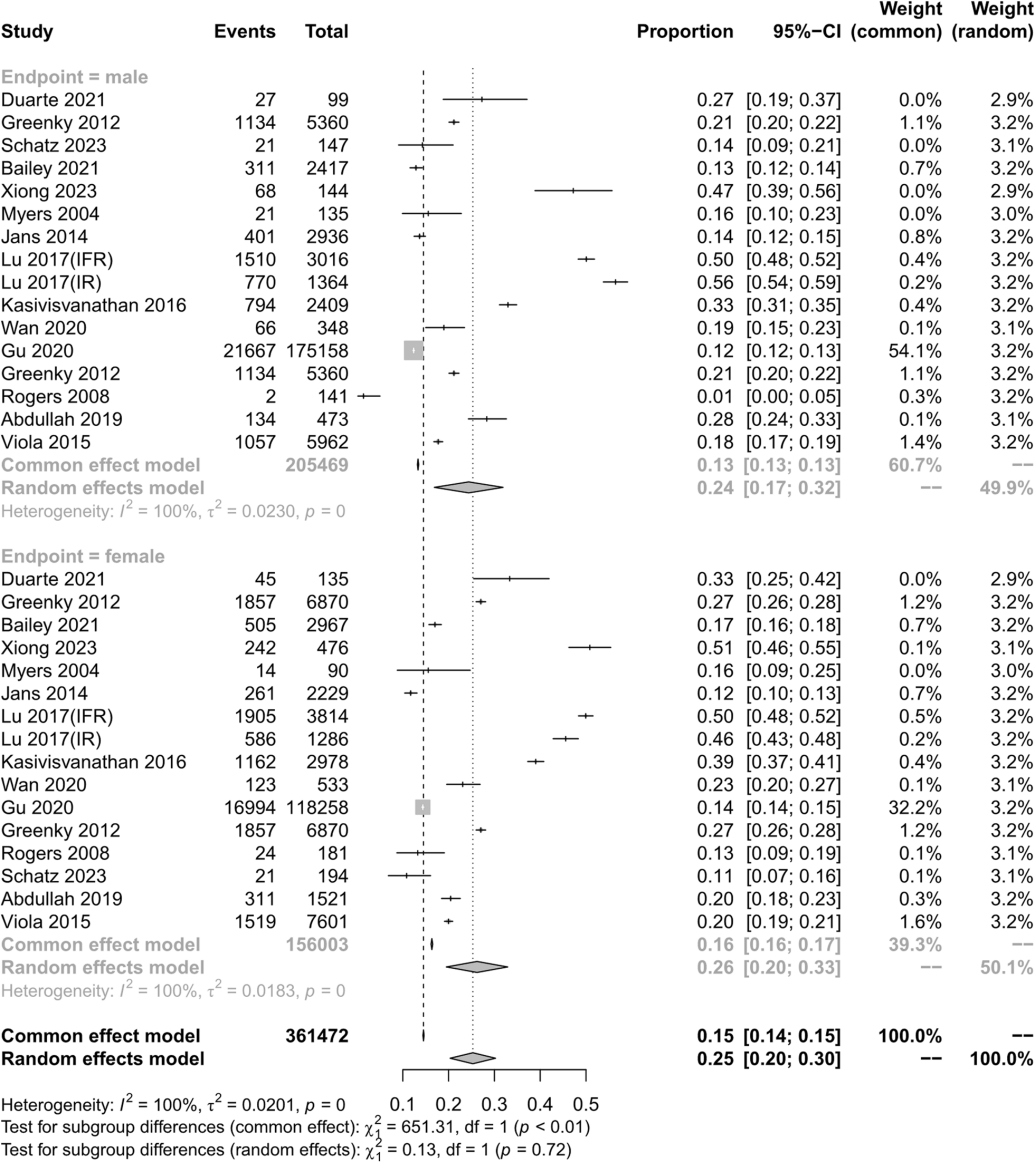
Prevalence of preoperative anaemia by gender

When we analyzed the prevalence of preoperative anemia according to sex, in males the prevalence was 22.8% [1, 14, 17, 24–26, 30–33, 37–40]. The prevalence of preoperative anemia was 22.8%, (95% CI 16.0–30.4%; *I*^2^ = 100%; *P* < 0.01) in males and 25.5%, (95% CI 19.5–32.1%; *I*^2^ = 100%; *P* < 0.01) in females [1, 14, 17, 24–26, 30–33, 37–40]. The prevalence was 25.5%, (95% CI 19.5–32.1%; *I*^2^ = 100%; *P* < *0.*01) (Fig. 5).

3. Preoperative comorbidities

1. Combined hypertension

**Fig. 6:**
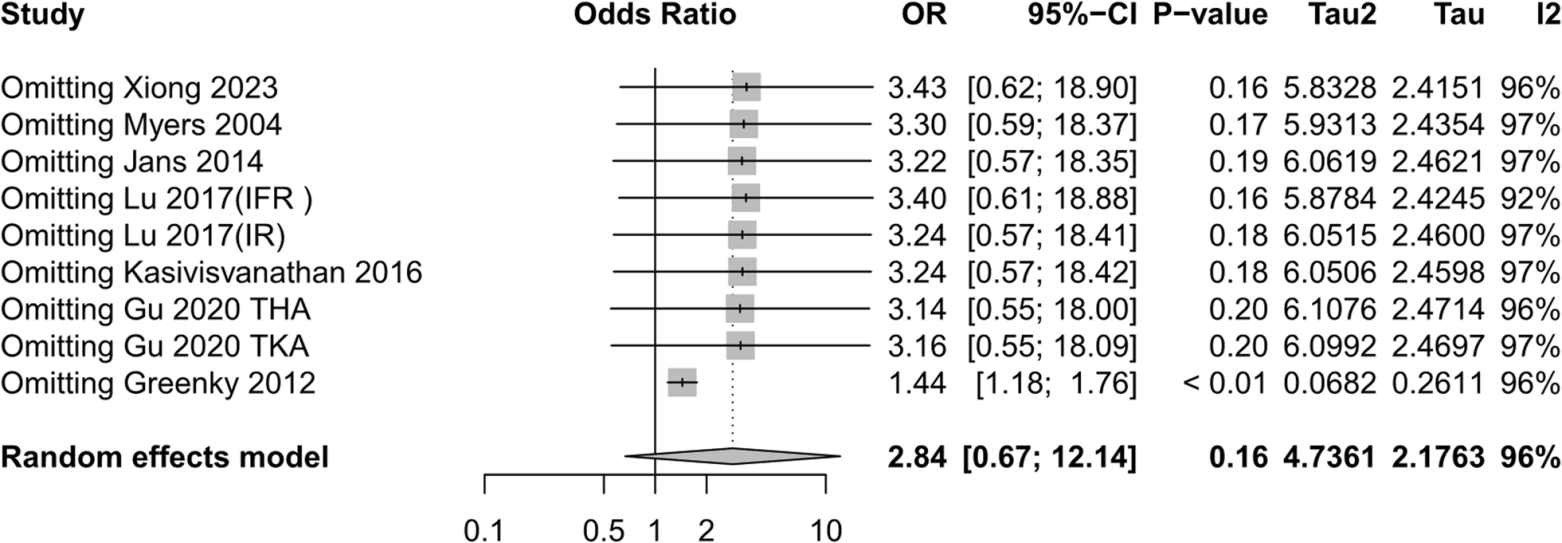
Prevalence of hypertension

Seven studies [1, 14, 17, 24, 25, 30, 31] reported preoperative anemic or non-anemic patients with preoperative comorbid hypertension. A random-effects model found a higher prevalence of hypertension in patients with preoperative anemia (RR = 1.26, 95% CI [1.04,1.53], *P* < 0.00001, *I*^*2*^ = 100%) (Fig. 6); however, this finding may be influenced by high heterogeneity (The results of sensitivity analysis are shown in Additional file 4: Annex 4).

(2) Combined diabetes mellitus

**Fig. 7:**
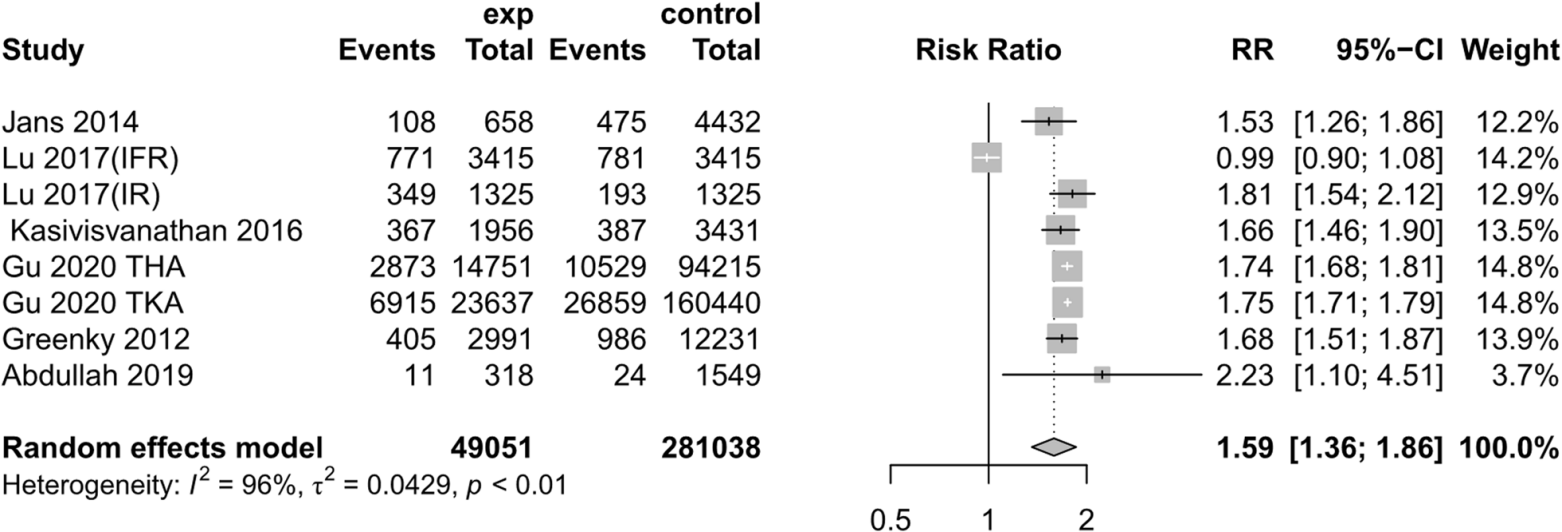
Prevalence of diabetes mellitus

Six studies [1, 14, 17, 24, 31, 33] reported preoperative anemic patients or preoperative non-anemic patients with preoperative comorbid diabetes, random-effects models suggested that preoperative anemia increased the risk of diabetic disease (RR = 1.59, 95%CI [1.36,1.86] *P* < 0.01, *I*^2^ = 96%) (Fig. 7). However, this finding may have been affected by high heterogeneity (The results of sensitivity analysis are shown in Additional file 4: Annex 4).

(3) Combined chronic obstructive pulmonary disease (COPD)

**Fig. 8:**
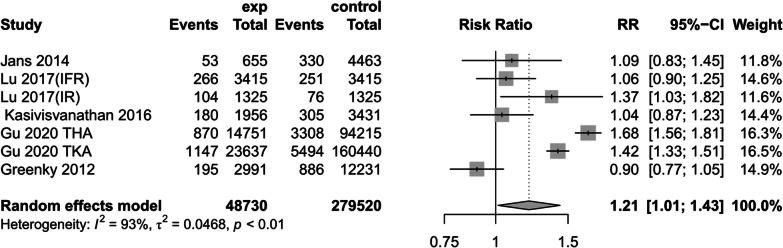
Prevalence of Chronic Obstructive Pulmonary Disease (COPD)

Five studies [1, 14, 17, 24, 31] investigated the incidence of preoperative anemic or preoperative non-anemic in patients with preoperative COPD comorbidities. A random-effects model revealed a difference in the increased incidence of COPD in patients with preoperative anemia (RR = 1.12, 95% CI [1.01, 1.43], *P* < 0.01, *I*^2^ = 93%) (Fig. 8)(The results of sensitivity analysis are shown in Additional file 4: Annex 4).

4. Postoperative clinical outcomes

1. Deep postoperative infections

**Fig. 9:**
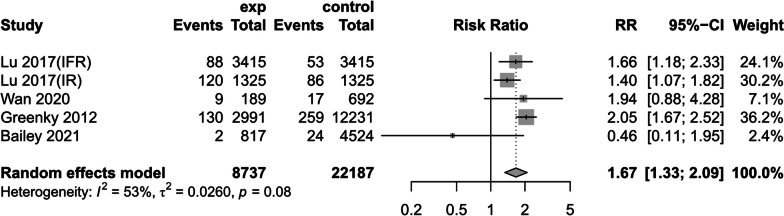
Postoperative deep infection

Four studies[14, 31, 38, 40] reported the effect of preoperative anemia on deep infection after primary lower-limb arthroplasty. Using a random-effects model, we observed an increased risk of deep infection in patients with preoperative anemia (RR = 1.67, 95% CI [1.33,2.09], *P* < 0.081, *I*^2^ = 53%) (Fig. 9).

(2) Postoperative superficial infection

**Fig. 10:**
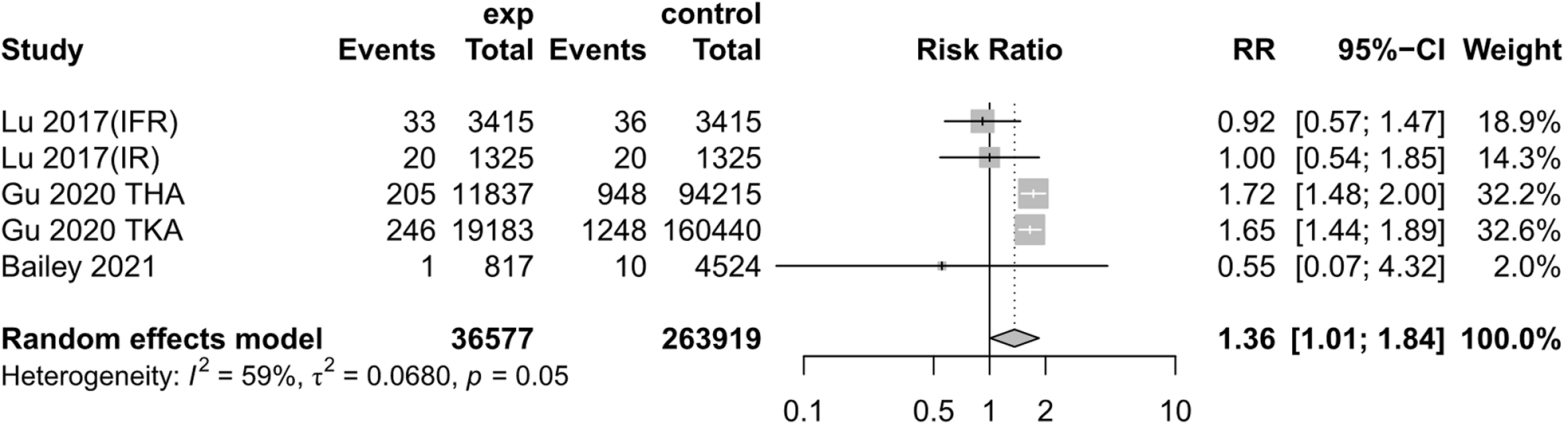
Postoperative superficial infection

Three studies [14, 17, 24]referred to the association between preoperative anemia and superficial infections and, using a random-effects model, suggested that preoperative anemia would increase the incidence of superficial infections (RR = 1.36, 95% CI [1.01, 1.84], *P* < 0.05, *I*^2^ = 59%) (Fig. 10).

(3) Post-operative blood transfusion rate.

**Fig. 11:**
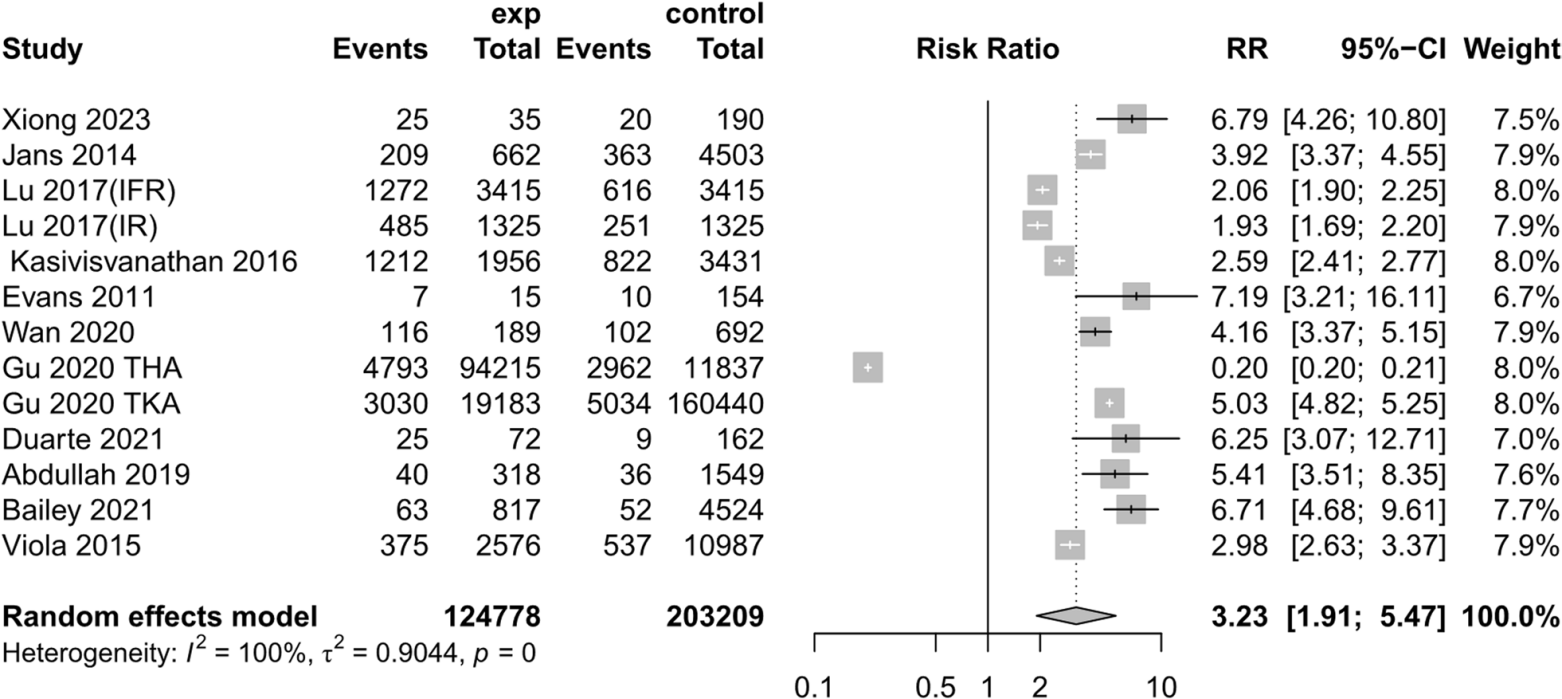
Postoperative blood transfusion rates

Eleven studies [1, 14, 17, 24, 30, 32, 33, 35, 38–40] reported a particularly significant difference in postoperative transfusion rates between preoperatively anemic and non-anemic patients (RR = 3.23, 95% CI [1.91,5.47], *P* < 0.00001, *I*^*2*^ = 100%) (Fig. 11) (The results of sensitivity analysis are shown in Additional file 4: Annex 4).

(4) Postoperative blood transfusion.

**Fig. 12:**
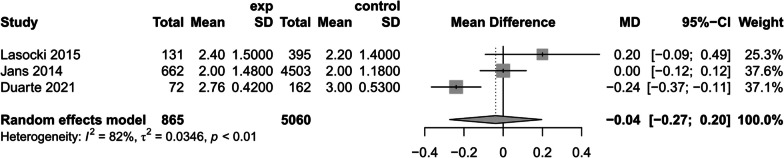
Postoperative blood transfusions

Three studies [1, 34, 39] mentioned the relationship between preoperative anemia and blood transfusion, using a random-effects model, suggested that preoperative anemia would increase the amount of blood transfused in postoperative patients (MD = − 0.04, 95% CI [− 0.27,0.20], *P* > 0.05, *I*^2^ = 82%) (Fig. 12). However, the number of inclusions and heterogeneity *I*^*2*^ were large, and this result should be viewed carefully.

(5) DVT

**Fig. 13:**
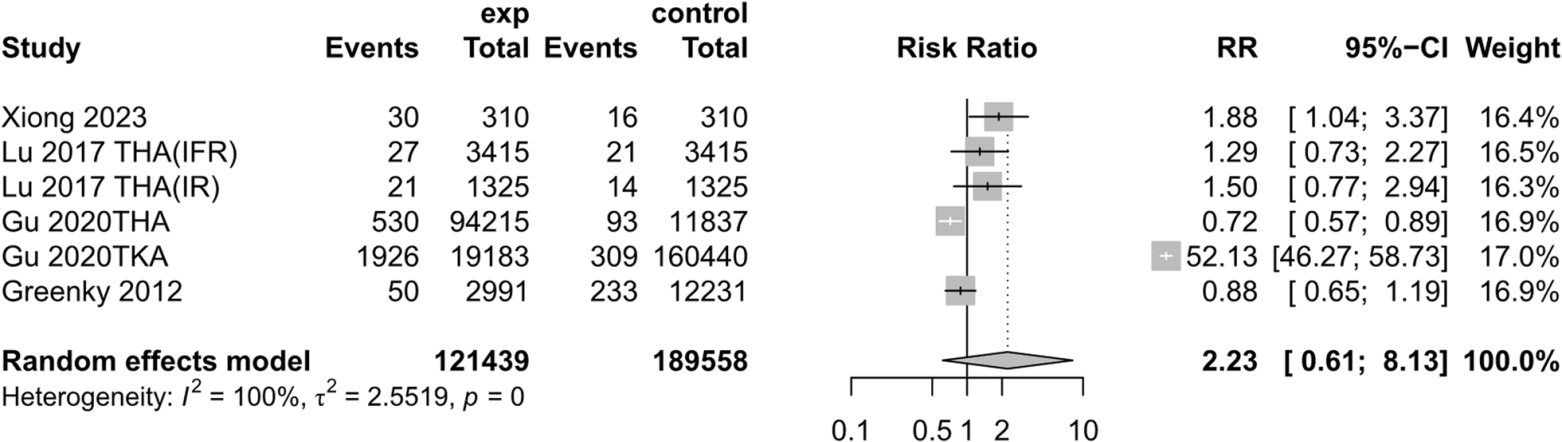
DVT

Four studies [14, 17, 30, 31] reported the effect of preoperative anemic versus non-anemic patients on postoperative DVT, using a random-effects model to create a forest plot showing that preoperative anemic patients had a 2.23-fold risk of DVT compared with preoperative non-anemic patients ( RR = 2.23, 95% CI [0.61, 8.13], *P* = 0.0001, *I*^2^ = 100%) (Fig. 13) (The results of sensitivity analysis are shown in Additional file 4Annex 4).

(6) Number of days in hospital

**Fig. 14:**
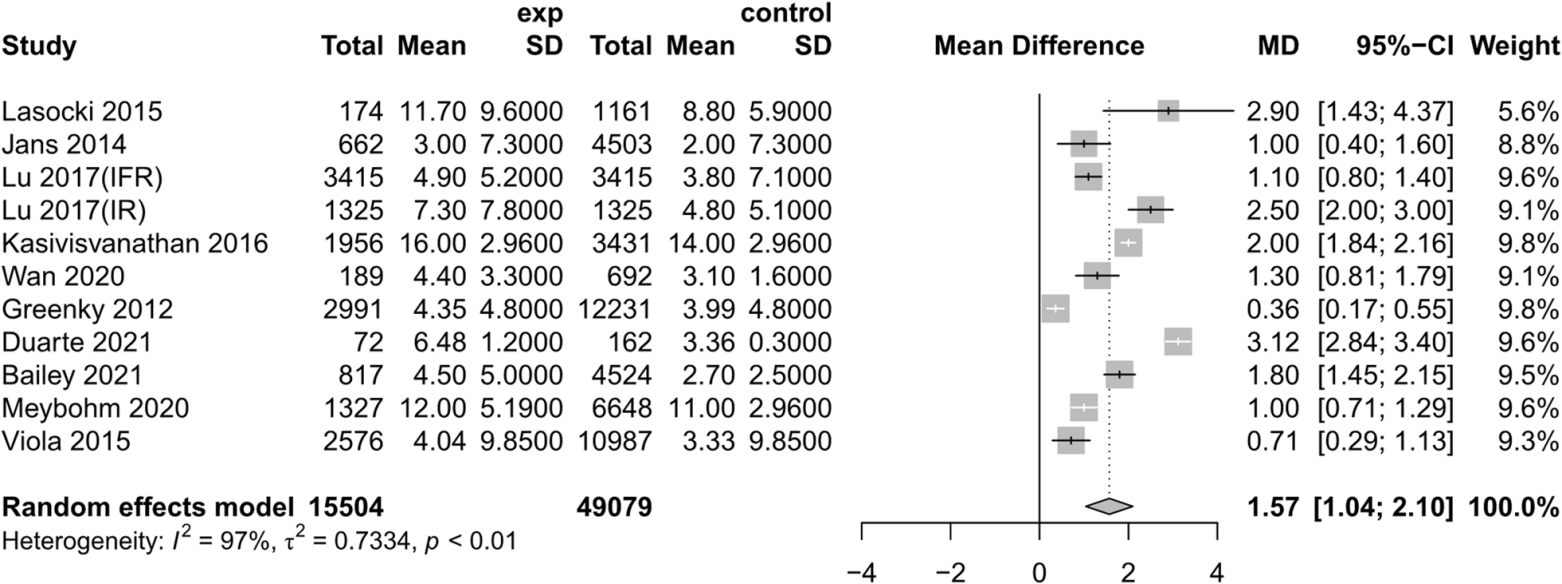
Forest plot of hospitalisation days

Ten studies [1, 14, 24, 31, 32, 34, 36, 38–40] reported the effect of preoperative anemic versus non-anemic patients on the number of days in the hospital, using a random-effects model to create a forest plot, and their results showed that preoperative anemic patients had increased the risk of patient days in hospital when compared with preoperative non-anemic patients (MD = 1.57, 95% CI [1.04, 2.10], *P* < 0.01, *I*^2^ = 97%,) (Fig. 14) (The results of sensitivity analysis are shown in Additional file 4Annex 4).

(7) Re-hospitalisation rate within three months

**Fig. 15:**
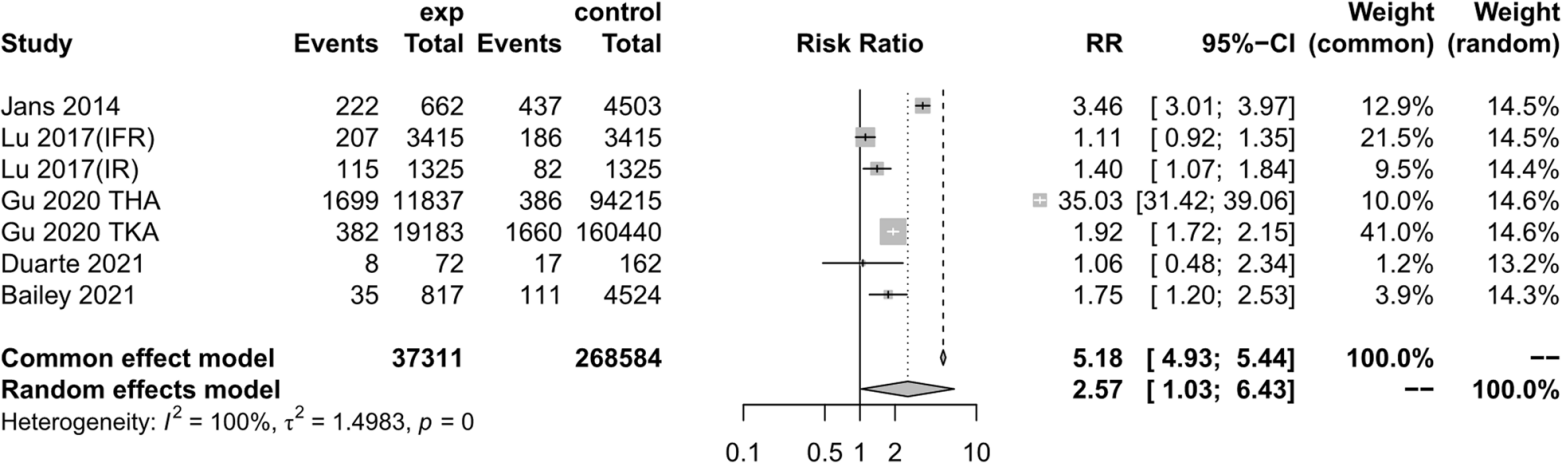
Forest plot of readmission rates within three months

Five studies reported on the effect of preoperative anemic versus non-anemic patients on readmission rates within 3 months [1, 14, 17, 38, 39], due to large heterogeneity (*I*^2^ = 100%) using a random-effects model to create a forest plot, the results of which showed that patients with preoperative anemia would be at significantly greater risk of readmission rates compared with patients with preoperative non-anemia (RR = 2.57, 95% CI [1.03, 6.43], *P* < 0.04, *I*^2^ = 100%) (Fig. 15) (The results of sensitivity analysis are shown in Additional file 4Annex 4).

(8) Mortality

**Fig. 16:**
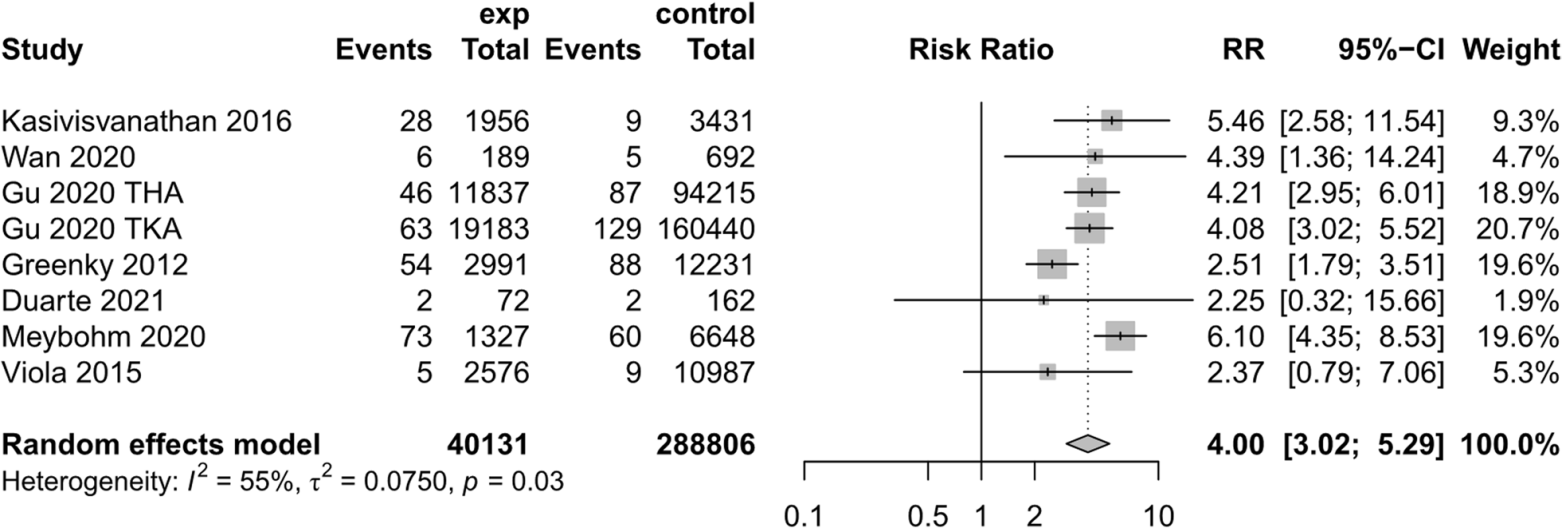
Mortality forest map

Seven studies [17, 24, 31, 32, 36, 39, 40] reported on the effect of preoperative anemic versus non-anemic patients on postoperative mortality. Because of the high heterogeneity (*I*^2^ = 55%), a random effects model was used to create a forest plot, the results of which showed that preoperative anemic patients would substantially increase the risk of mortality in patients compared with preoperative non-anemic patients (RR = 4.00, 95% CI [3.02, 5.29], *P* = 0.03) (Fig. 16) (The results of sensitivity analysis are shown in Additional file 4Annex 4).

## Other patient-reported outcomes

Two studies [25, 35] reported common causes of preoperative anemia; however, they were not combined because of the small number of included studies and missing standard deviations because the studies did not provide complete data. Therefore, only a systematic evaluation of the common causes of preoperative anemia was performed, and both studies came to a similar conclusion that the most common cause of preoperative anemia was iron deficiency anemia and that preoperative treatment of iron deficiency anemia was associated with an improved prognosis and a reduction in allogeneic transfusions.

### Grading of evidence

The overall quality of evidence was low given the observational design of the included studies. Evidence for all outcomes was of low quality owing to the stage of the study, imprecision of effect sizes, study limitations, inconsistency, or publication bias (Table 3).

**Table 3:** Quality of evidence

Outcomes	Limitations of the study	Inconsistency I^2^ > 50 per cent	Indirectness Yes: ↓	inaccuracy	Publication bias Yes or unclear: ↓	Effect size RR (95% CI) Lower limit RR > 2.0: ↑	Overall quality of evidence
Complicated with hypertension	1 ↓	*I*^*2*^ = 100% ↓	No	No	No	RR = 1.26,95 % CI [1.04,1.53]	Low
Complicated with diabetes mellitus	1 ↓	I^2^ = 96% ↓	No	No	No	RR = 1.59,95 % CI [1.36,1.86]	Low
Complicated with COPD	1 ↓	I^2^ = 93% ↓	No	No	No	RR = 1.12, 95 % CI [1.01,1.43]	Low
Postoperative deep infection	1 ↓	I^2^ = 53% ↓	No	No	No	RR = 1.67, 95 % CI [1.33,1.95]	Low
Postoperative superficial infection	1 ↓	I^2^ = 59% ↓	No	No	No	RR = 1.36, 95 % CI [1.01,1.84]	Low
Postoperative blood transfusion rate	1 ↓	I^2^ = 100% ↓	No	No	No	RR = 1.36, 95 % CI [1.01,1.84]	Low
Postoperative blood transfusion volume	1 ↓	I^2^ = 82% ↓	No	No	No	MD = -0.04, 95 % CI [-0.27,0.20]	Low
DVT	1 ↓	I^2^ = 100% ↓	No	Yes ↓	No	RR = 2.23, 95 % CI [0.61, 8.13] ↑	Low
Hospital days	1 ↓	I^2^ = 97 per cent ↓	No	No	No	MD = 1.66, 95 % CI [1.10, 2.21]	Low
Rehospitalisation rate within three months	1 ↓	I^2^ = 100% ↓	No	Yes ↓	No	RR = 2.57, 95 % CI [1.03, 6.42] ↑	Low
mortality	1 ↓	I^2^ = 59% ↓	No	Yes ↓	No	RR = 4.12, 95 % CI [3.08, 5.51] ↑	Low

*GRADE* grading of recommendations assessment development and evaluation; *RR* relative risk; *MD* mean difference

↓ signifies a downgrade in quality of evidence; ↑ signifies an upgrade in quality of evidence

## Discussion

Preoperative anemia is a common and significant risk factor for adverse events after joint replacement. Anemia affects up to 21–35% of patients undergoing primary or revision total joint arthroplasty [4, 32, 41]. The association between preoperative anemia and postoperative complications such as infection, mortality, length of hospital stay, and functional status has been reported in several studies [25, 42–44].

In this study, we found an overall prevalence of preoperative anemia of 22%, which varied considerably by type of surgery, with the highest prevalence of preoperative anemia of 38.3% in patients with TKA awaiting revision, and the lowest prevalence of preoperative anemia of 15.2% in patients undergoing first-time hip arthroplasty. The lowest prevalence was 17.3 percent in Europe, with similar rates in Asia and the Americas (26.6–28.1%).

Gender is an immutable factor for total joint arthroplasty waiting for patients with preoperative anemia. We found that the prevalence of preoperative anemia was slightly higher in women (25.5%) than in men (22.8%) undergoing total joint arthroplasty. There are multiple reasons for this phenomenon. In addition to the lack of androgens in women compared with men, due to the physiological cycle, the different distribution of factors such as diabetes and metabolic syndrome between the sexes may also contribute to this inconsistency [45–47].

To determine the impact of preoperative anemia on patients' surgical outcomes, we summarized previous studies and found that the available studies reported only a limited number (two or three) of crude outcome indicators (e.g., deaths, complications, infections, and myocardial infarction) [32, 34, 39]. Therefore, eight commonly used postoperative evaluation indicators were included in this study to achieve a more detailed and adequate measure of the impact of preoperative anemia on patients' postoperative outcomes.

Comorbidities are common in patients with preoperative anemia, and the majority of older people awaiting joint replacement have three or more comorbidities [14, 17, 24]. Our meta-analysis found statistically significant differences in several preoperative comorbidities (hypertension, diabetes mellitus, and COPD) between the preoperative anemic and preoperative non-anemic groups. However, the results require careful consideration because of the high degree of heterogeneity observed in comorbidity analyses. We performed sensitivity analyses for comorbidities to identify sources of high heterogeneity and found that studies of comorbid hypertension versus comorbid COPD were relatively stable; however, when we performed sensitivity analyses for patients with comorbid diabetes, we found that by removing Lu 2017 (IFR) et al. [14], the heterogeneity was reduced to 0%. Analyzing possible reasons compared to other included studies, Lu 2017 (IFR) et al. [14] performed propensity score matching to control for selection bias, and this article was the only one to show that the risk of preoperative anemia combined with diabetes mellitus was lower than the risk of preoperative non-anemia combined with diabetes mellitus.

Surgical site infection (SSI) is the most common complication. Deep infection around the prosthesis is one of the most serious orthopedic complications in patients and will increase readmission and mortality rates [14, 31, 48, 49]. Our study showed that superficial and deep infections occurred with equal frequency in preoperatively anemic patients, suggesting that preoperative anemic patients have poorer immunity than preoperative non-anemic patients. Therefore, rational use of antibiotics and strict asepsis during the perioperative period in preoperatively anemic patients is essential.

In this study, we assessed the effect of preoperative anemia on postoperative transfusion rates and volumes in patients undergoing elective hip and knee arthroplasty. Our findings are consistent with the published literature showing that preoperative anemia negatively affects individual transfusion risk [1, 14, 24, 39]. Meta-analysis of the study showed that the preoperative anemic group had a higher risk of postoperative transfusion rate than the preoperative non-anemic group (RR = 3.23), but there was no statistically significant difference in the volume of transfusion between the two groups postoperatively; however, the number of inclusions and heterogeneity *I*^2^ was large, and this result should be viewed carefully. In addition, although transfusions are undoubtedly necessary for patients with acute anemia, they have deleterious effects, including transfusion-associated lung injury, hospital-acquired infections, volume overload, immunomodulation, and delayed physiotherapy in transfusion recipients [50, 51]. Finally, preoperative anemia reduces physiological oxygen-carrying capacity, which in turn impairs other organ systems such as cardiac perfusion, lung function, and wound healing, and blood, an increasingly scarce product that is dependent on voluntary donors, must be used in a restrictive and rational manner with attention to the need to treat anemia preoperatively.

DVTof the lower extremities is a common complication after joint replacement surgery. Iron deficiency anemia is an independent predictor of VTE recurrence in patients with unexplained thrombosis [52], and patients are at high risk of VTE after joint replacement surgery [53]. In the absence of pharmacological intervention, the incidence of asymptomatic deep vein thrombosis after TKA ranges from 40 to 85%, and the incidence of fatal PE ranges from 0.87 to 1.99% [54]. Preoperative anemia has been reported to increase blood volume and blood substances [55–58]. Therefore, patients with preoperative anemia must be thoroughly investigated and hypercoagulability controlled when undergoing TKA or THA. Arranging relevant anticoagulation therapy and encouraging patients to exercise early may reduce the recurrence of DVT and serious complications.

We found that preoperative anemia was associated with length of hospital stay and readmission rates. The more severe the anemia, the longer the hospital stay, the worse the outcome, and the higher the cost. International guidelines recommend early detection of preoperative anemia, identification of the cause, and treatment of any potentially reversible causes, such as iron deficiency. Treatment of anemia has been shown to reduce postoperative blood transfusions, length of hospital stay, and 30-day readmission rates. A previous study reported that preoperative intravenous iron treatment of iron deficiency anemia in patients undergoing major abdominal surgery reduced the median hospital stay by 3 days [59]. Similar results have been achieved with elective lower limb arthroplasty in the United Kingdom and Australia [60]. Abdullah et al. showed that each 1-g increase in preoperative Hb reduced the patient's hospital stay by 0.2 days. It is therefore necessary to go further and examine the range of postoperative effects of preoperative treatment of anemia, and again the importance of preoperative treatment of anemia [33].

To the best of our knowledge, the effect of preoperative anemia on mortality in patients undergoing orthopedic surgery has been controversial in recent years. In our meta-analysis, forest plots showed 389 deaths out of 288,806 patients in the preoperative non-anemic group, a mortality rate of 0.13%, compared with 277 deaths out of 40,131 patients in the preoperative anemic group, a mortality rate of 0.7%. The difference in mortality after lower limb arthroplasty between the two groups was significant, with preoperative anemia having a 4.00 times risk of mortality compared to preoperative non-anemic patients. However, our findings on mortality should be watched carefully because of the large heterogeneity in results (*I*^2^ = 55%).

Age is also a widely accepted risk factor for preoperative anemia. Large-population studies have shown that the prevalence of preoperative anemia increases with age [61–63]. Our preliminary study did not report prevalence rates in different age groups; therefore, we were unable to pool the prevalence rates by age subgroups. Thus, age may have been a source of the heterogeneity in our study. Future studies of patients with TJA are encouraged to report age-specific prevalence rates. As many patients over 65 years of age undergo TJA, it would be an interesting study to explore the effect of preoperative anemia on the postoperative period of arthroplasty in this or older age group [63].

To our knowledge, this is the first meta-analysis to assess the impact of preoperative anemia on TJA outcomes. The strength of this study is that it provides evidence from an evidence-based medical perspective to reinforce surgeons' caution in approaching preoperative anemia after it has been fully demonstrated that preoperative anemia has several detrimental effects on waiting for arthroplasty. Furthermore, our literature search was comprehensive, enabling us to make meaningful estimates of the impact of the clinically significant outcomes. However, this study has several limitations. First, this study included only 21 retrospective, single-center, or multicenter studies, some of which were small-sample studies, and it suffers from the standard bias of this type of study. Second, the period of the study was either long or short, and some important practical changes may have occurred during this period. These changes may have led to the biases associated with the assessment. Third, the overall heterogeneity of the studies was high, which may be due to differences in sample size, study population, mean age, sex differences, and experimental methods of individual studies. Therefore, this study may have had an assessment-related bias.

## Summary

Preoperative anemia is common in patients awaiting THR or TKR. The prevalence of preoperative anemia is 22% and is associated with poorer postoperative outcomes and increased transfusion volumes and rates. This study confirms that anemia is an independent factor associated with poor outcomes after primary or revision arthroplasty. Therefore, surgeons should strongly consider preoperative, timely, and optimal anemia, as it may reduce the "anemia-related negative outcomes" after arthroplasty.

### Supplementary Information

**Additional file 1. Annex 1** Search formulate.

**Additional file 2. Annex 2** Literature screening flow chart.

**Additional file 3. Annex 3** Kappa.

**Additional file 4. Annex 4** Sensitivity analysis and Egger.
