# Supplementary material for: Impact of preoperative anemia on patients undergoing total joint replacement of lower extremity: a systematic review and meta-analysis
Source: J Orthop Surg Res. 2024 Apr 18;19:249. doi: 10.1186/s13018-024-04706-y (PMC11027536; doi:10.1186/s13018-024-04706-y)
Supplement: Supplementary file 1 — Additional file 1. Annex 1 Search formulate. [file 13018_2024_4706_MOESM1_ESM.docx]

Pubmed 234

((((Arthroplasties, Replacement, Hip[Title/Abstract] OR Arthroplasty, Hip Replacement[Title/Abstract] OR Hip Replacement Arthroplasties[Title/Abstract] OR Hip Prosthesis Implantation[Title/Abstract] OR Hip Prosthesis Implantations[Title/Abstract] OR Implantation, Hip Prosthesis[Title/Abstract] OR Prosthesis Implantation, Hip[Title/Abstract] OR Replacement Arthroplasties, Hip[Title/Abstract] OR Replacement Arthroplasty, Hip[Title/Abstract] OR Arthroplasties, Hip Replacement[Title/Abstract] OR Hip Replacement Arthroplasty[Title/Abstract] OR Hip Replacement, Total[Title/Abstract] OR Replacement, Total Hip[Title/Abstract] OR Total Hip Replacements[Title/Abstract] OR Total Hip Replacement[Title/Abstract] OR Total Hip Arthroplasty[Title/Abstract] OR Arthroplasty, Total Hip[Title/Abstract] OR Hip Arthroplasty, Total[Title/Abstract] OR Total Hip Arthroplasties[Title/Abstract] OR "Arthroplasty, Replacement, Hip"[Mesh])) OR ((Arthroplasties, Replacement, Knee[Title/Abstract] OR Arthroplasty, Knee Replacement[Title/Abstract] OR Knee Replacement Arthroplasties[Title/Abstract] OR Knee Replacement Arthroplasty[Title/Abstract] OR Replacement Arthroplasties, Knee[Title/Abstract] OR Knee Arthroplasty, Total[Title/Abstract] OR Arthroplasty, Total Knee[Title/Abstract] OR Total Knee Arthroplasty[Title/Abstract] OR Replacement, Total Knee[Title/Abstract] OR Total Knee Replacement[Title/Abstract] OR Knee Replacement, Total[Title/Abstract] OR Knee Arthroplasty[Title/Abstract] OR Arthroplasty, Knee[Title/Abstract] OR Arthroplasties, Knee Replacement[Title/Abstract] OR Replacement Arthroplasty, Knee[Title/Abstract] OR knee joint replacement OR knee joint replacements OR "Arthroplasty, Replacement, Knee"[Mesh])))) AND ((( (preoperative care)[Title/Abstract] OR (perioperative period)[Title/Abstract] OR (perioperative care)[Title/Abstract]OR (pre operatively)[Title/Abstract] OR ("Preoperative Period"[Mesh])) AND ((Anemias)[Title/Abstract] OR ("Anemia"[Mesh])))

embass: 33

'preoperative period':ab,ti OR 'preoperative care':ab,ti OR 'perioperative period':ab,ti OR 'perioperative care':ab,ti OR 'pre operatively':ab,ti

'Anemias':ab,ti OR 'Anemia':ab,ti

arthroplasties,:ti,ab,kw AND replacement,:ti,ab,kw AND knee:ti,ab,kw OR (arthroplasty,:ti,ab,kw AND knee:ti,ab,kw AND replacement:ti,ab,kw) OR (knee:ti,ab,kw AND replacement:ti,ab,kw AND arthroplasties:ti,ab,kw) OR (knee:ti,ab,kw AND replacement:ti,ab,kw AND arthroplasty:ti,ab,kw) OR (replacement:ti,ab,kw AND arthroplasties,:ti,ab,kw AND knee:ti,ab,kw) OR (knee:ti,ab,kw AND arthroplasty,:ti,ab,kw AND total:ti,ab,kw) OR (arthroplasty,:ti,ab,kw AND total:ti,ab,kw AND knee:ti,ab,kw) OR (total:ti,ab,kw AND knee:ti,ab,kw AND arthroplasty:ti,ab,kw) OR (replacement,:ti,ab,kw AND total:ti,ab,kw AND knee:ti,ab,kw) OR (total:ti,ab,kw AND knee:ti,ab,kw AND replacement:ti,ab,kw) OR (knee:ti,ab,kw AND replacement,:ti,ab,kw AND total:ti,ab,kw) OR (knee:ti,ab,kw AND arthroplasty:ti,ab,kw) OR (arthroplasty,:ti,ab,kw AND knee:ti,ab,kw) OR (arthroplasties,:ti,ab,kw AND knee:ti,ab,kw AND replacement:ti,ab,kw) OR (replacement:ti,ab,kw AND arthroplasty,:ti,ab,kw AND knee:ti,ab,kw) OR (knee:ti,ab,kw AND joint:ti,ab,kw AND replacement:ti,ab,kw) OR (knee:ti,ab,kw AND joint:ti,ab,kw AND replacements:ti,ab,kw)

'arthroplasties, replacement, hip':ab,ti OR 'arthroplasty, hip replacement':ab,ti OR 'hip replacement arthroplasties':ab,ti OR 'hip prosthesis implantation':ab,ti OR 'hip prosthesis implantations':ab,ti OR 'implantation, hip prosthesis':ab,ti OR 'prosthesis implantation, hip':ab,ti OR 'replacement arthroplasties, hip':ab,ti OR 'replacement arthroplasty, hip':ab,ti OR 'arthroplasties, hip replacement':ab,ti OR 'hip replacement arthroplasty':ab,ti OR 'hip replacement, total':ab,ti OR 'replacement, total hip':ab,ti OR 'total hip replacements':ab,ti OR 'total hip replacement':ab,ti OR 'total hip arthroplasty':ab,ti OR 'arthroplasty, total hip':ab,ti OR 'hip arthroplasty, total':ab,ti OR 'total hip arthroplasties':ab,ti OR 'arthroplasty, replacement, hip':ab,ti

WEB OF SICENCE 203

((((((((((((((((((TS=(Arthroplasty, Replacement, Knee)) OR TS=(Arthroplasties, Replacement, Knee)) OR TS=(Arthroplasty, Knee Replacement)) OR TS=(Knee Replacement Arthroplasties)) OR TS=(Knee Replacement Arthroplasty)) OR TS=(Replacement Arthroplasties, Knee)) OR TS=(Knee Arthroplasty, Total)) OR TS=(Arthroplasty, Total Knee)) OR TS=(Total Knee Arthroplasty)) OR TS=(Replacement, Total Knee)) OR TS=(Total Knee Replacement)) OR TS=(Knee Replacement, Total)) OR TS=(Knee Arthroplasty)) OR TS=(Arthroplasty, Knee)) OR TS=(Arthroplasties, Knee Replacement)) OR TS=(Replacement Arthroplasty, Knee)) OR TS=(knee joint replacement)) OR TS=(knee joint replacements)) OR TS=(Replacement Arthroplasty, Knee)

(((((((((((((((((((TS=(Arthroplasties, Replacement, Hip)) OR TS=(Arthroplasty, Hip Replacement)) OR TS=(Hip Replacement Arthroplasties)) OR TS=(Hip Prosthesis Implantation)) OR TS=(Hip Prosthesis Implantations)) OR TS=( Implantation, Hip Prosthesis)) OR TS=(Prosthesis Implantation, Hip)) OR TS=(Replacement Arthroplasties, Hip)) OR TS=(Replacement Arthroplasty, Hip)) OR TS=(Arthroplasties, Hip Replacement)) OR TS=( Hip Replacement Arthroplasty)) OR TS=(Hip Replacement, Total)) OR TS=(Replacement, Total Hip)) OR TS=(Total Hip Replacements)) OR TS=(Total Hip Replacement)) OR TS=(Total Hip Arthroplasty)) OR TS=(Arthroplasty, Total Hip)) OR TS=(Hip Arthroplasty, Total)) OR TS=(Total Hip Arthroplasties)) OR TS=(Arthroplasty, Replacement, Hip)

((((TS=(preoperative period)) OR TS=(pre operatively)) OR TS=('preoperative care))OR TS=(perioperative period)) OR TS=(perioperative care)

(TS=(Anemias)) OR TS=(Anemia)

COCHRAN 14

(Arthroplasty, Replacement, Knee):ti,ab,kw OR (Arthroplasties, Replacement, Knee):ti,ab,kw OR (Arthroplasty, Knee Replacement):ti,ab,kw OR (Knee Replacement Arthroplasties):ti,ab,kw OR (Knee Replacement Arthroplasty):ti,ab,kw OR (Replacement Arthroplasties, Knee):ti,ab,kw OR (Knee Arthroplasty, Total):ti,ab,kw OR (Arthroplasty, Total Knee):ti,ab,kw OR (Total Knee Arthroplasty):ti,ab,kw OR (Replacement, Total Knee):ti,ab,kw OR (Total Knee Replacement):ti,ab,kw OR (Knee Replacement, Total):ti,ab,kw OR (Knee Arthroplasty):ti,ab,kw OR (Arthroplasty, Knee):ti,ab,kw OR (Arthroplasties, Knee Replacement):ti,ab,kw OR (Replacement Arthroplasty, Knee):ti,ab,kw OR (knee joint replacement):ti,ab,kw OR (knee joint replacements):ti,ab,kw

MeSH descriptor: [Arthroplasty, Replacement, Knee] explode all trees

(Arthroplasties, Replacement, Hip):ti,ab,kw OR (Arthroplasty, Hip Replacement):ti,ab,kw OR (Hip Replacement Arthroplasties):ti,ab,kw OR (Hip Prosthesis Implantation):ti,ab,kw OR (Hip Prosthesis Implantations):ti,ab,kw OR ( Implantation, Hip Prosthesis):ti,ab,kw OR (Prosthesis Implantation, Hip):ti,ab,kw OR (Replacement Arthroplasties, Hip):ti,ab,kw OR (Replacement Arthroplasty, Hip):ti,ab,kw OR (Arthroplasties, Hip Replacement):ti,ab,kw OR ( Hip Replacement Arthroplasty):ti,ab,kw OR (Hip Replacement, Total):ti,ab,kw OR (Replacement, Total Hip):ti,ab,kw OR (Total Hip Replacements):ti,ab,kw OR (Total Hip Replacement):ti,ab,kw OR (Total Hip Arthroplasty):ti,ab,kw OR (Arthroplasty, Total Hip):ti,ab,kw OR (Hip Arthroplasty, Total):ti,ab,kw OR (Total Hip Arthroplasties):ti,ab,kw

MeSH descriptor: [Arthroplasty, Replacement, Hip] explode all trees

(Anemias):ab,ti OR (Anemia):ab,ti

(preoperative period):ab,ti OR (pre operatively):ab,ti
