# Supplementary material for: Impact of preoperative anemia on patients undergoing total joint replacement of lower extremity: a systematic review and meta-analysis
Source: J Orthop Surg Res. 2024 Apr 18;19:249. doi: 10.1186/s13018-024-04706-y (PMC11027536; doi:10.1186/s13018-024-04706-y)
Supplement: Supplementary file 2 — Additional file 2. Annex 2 Literature screening flow chart. [file 13018_2024_4706_MOESM2_ESM.docx]

Databases were searched by computer : PUBMED ( n = 315), EMBASE ( n = 33 ), COCHRANE LIBRARY

( n = 14 ), WEB OF SICENCE( n = 203 )

(n= 565）

## Identification

Records after duplicates removed
(n =267)

Records excluded
(n = 298)

## Screening

Records screened
(n =267)

Literature without group comparison, unrelated studies and reviews were excluded.

(n = 206)

Full-text articles assessed for eligibility
(n =61)

## Eligibility

1.Read the full text and exclude inappropriate literature : ( n = 9 )

2.There was no comparable data between preoperative anemia group and non-anemia group : ( n = 8 )

3.Data were incomplete or could not be extracted : ( n = 10 )

4.The full text cannot be obtained : ( n = 13 )

Studies included in qualitative synthesis
(n =21)

Studies included in quantitative synthesis
(n =21)

## Included

**Fig.1** Preferred reporting items of systematic reviews and meta-analysis (PRISMA) flow diagram
