# Supplementary material for: Impact of preoperative anemia on patients undergoing total joint replacement of lower extremity: a systematic review and meta-analysis
Source: J Orthop Surg Res. 2024 Apr 18;19:249. doi: 10.1186/s13018-024-04706-y (PMC11027536; doi:10.1186/s13018-024-04706-y)
Supplement: Supplementary file 4 — Additional file 4. Annex 4 Sensitivity analysis and Egger. [file 13018_2024_4706_MOESM4_ESM.docx]

Sensitivity analysis and Egger 's test of some indicators.

(1)Combined hypertension.

Sensitivity analysis of preoperative hypertension revealed that Greenkly et al.(28) after deletion. Heterogeneity was reduced to 97%, and sensitivity analysis and reanalysis using a random-effects model reiterated this significant difference (RR = 1.16,95% CI:[1.08,1.25], P < 0.01, I2 = 97%) (Fig. 1). However, heterogeneity remains when considering this result.

| 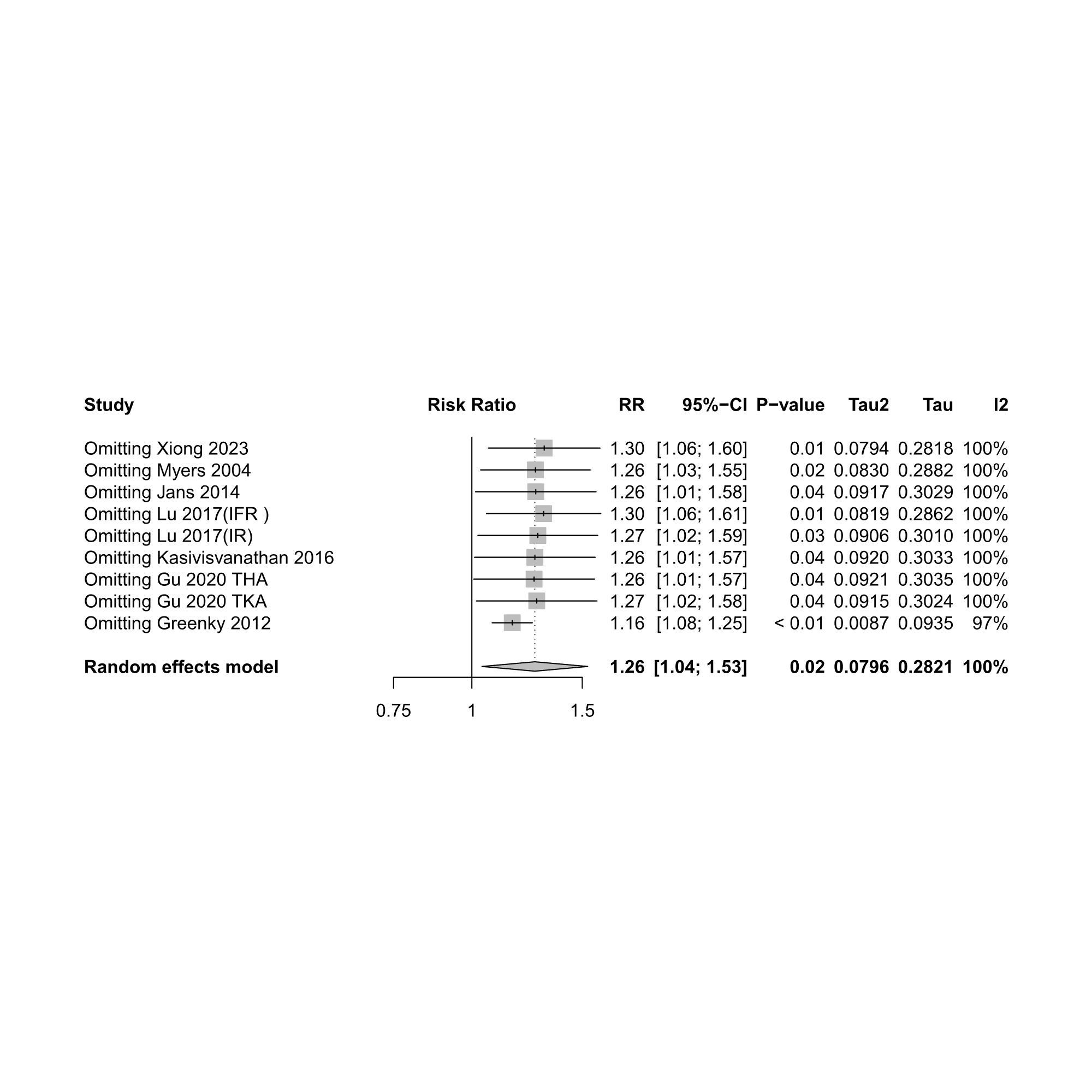 |
| --- |
| Fig. 1 |

(2)Combined diabetes mellitus

When sensitivity analyses were performed, after removing Lu 2017 (IFR) et al. from the study(14) , the heterogeneity was reduced to 0%, and reanalysis using a random-effects model reiterated that preoperative anemia significantly increased the prevalence of diabetes mellitus awaiting total joint replacement (RR = 1.74 ,95% CI:[1.71, 1.77], P < 0.0001, I2 = 0%) (Fig 2 ).

| 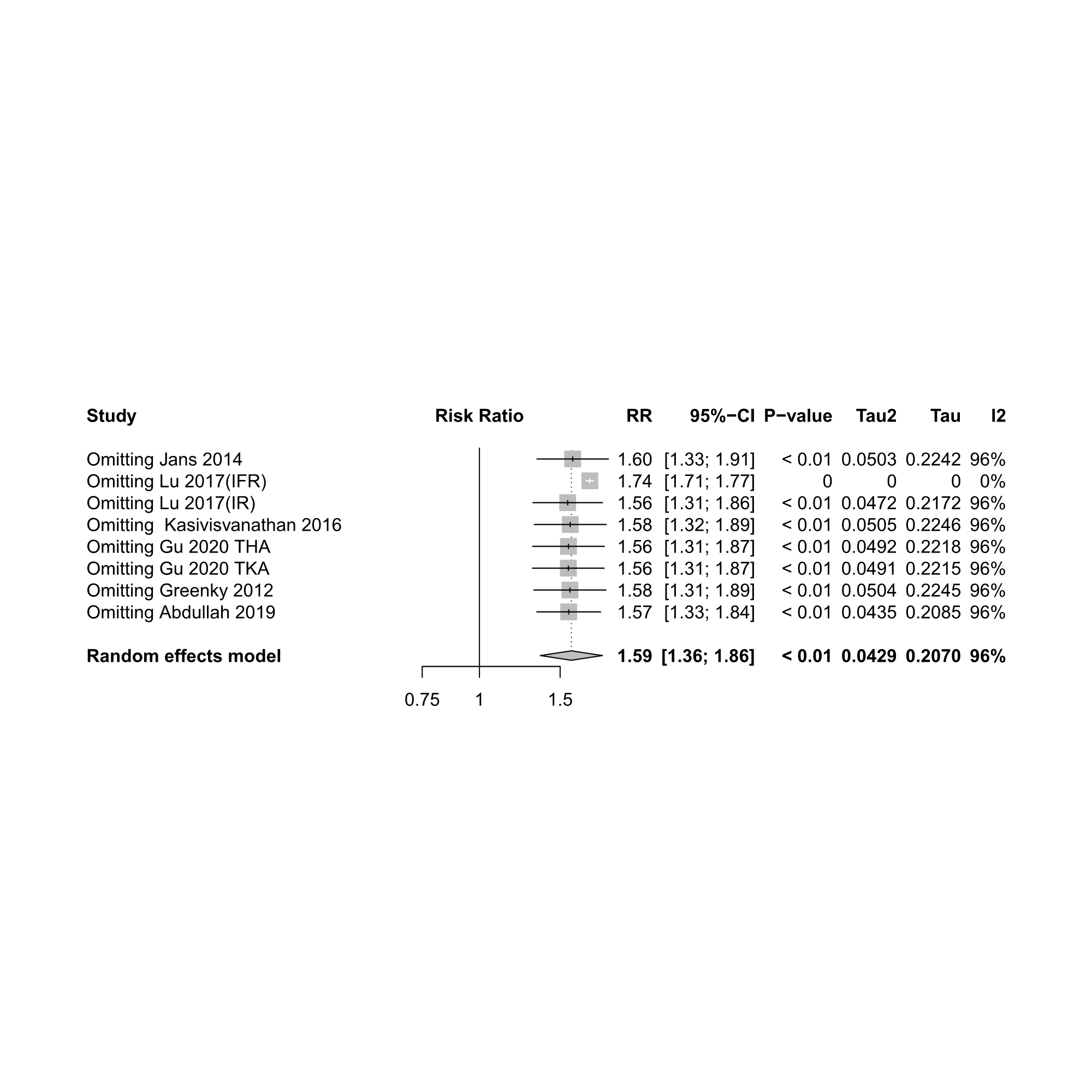 |
| --- |
| Fig 2 |

(3)Combined Chronic Obstructive Pulmonary Disease (COPD)

Sensitivity analysis of anemia combined with COPD before operation showed no significant change(Fig. 3).

| 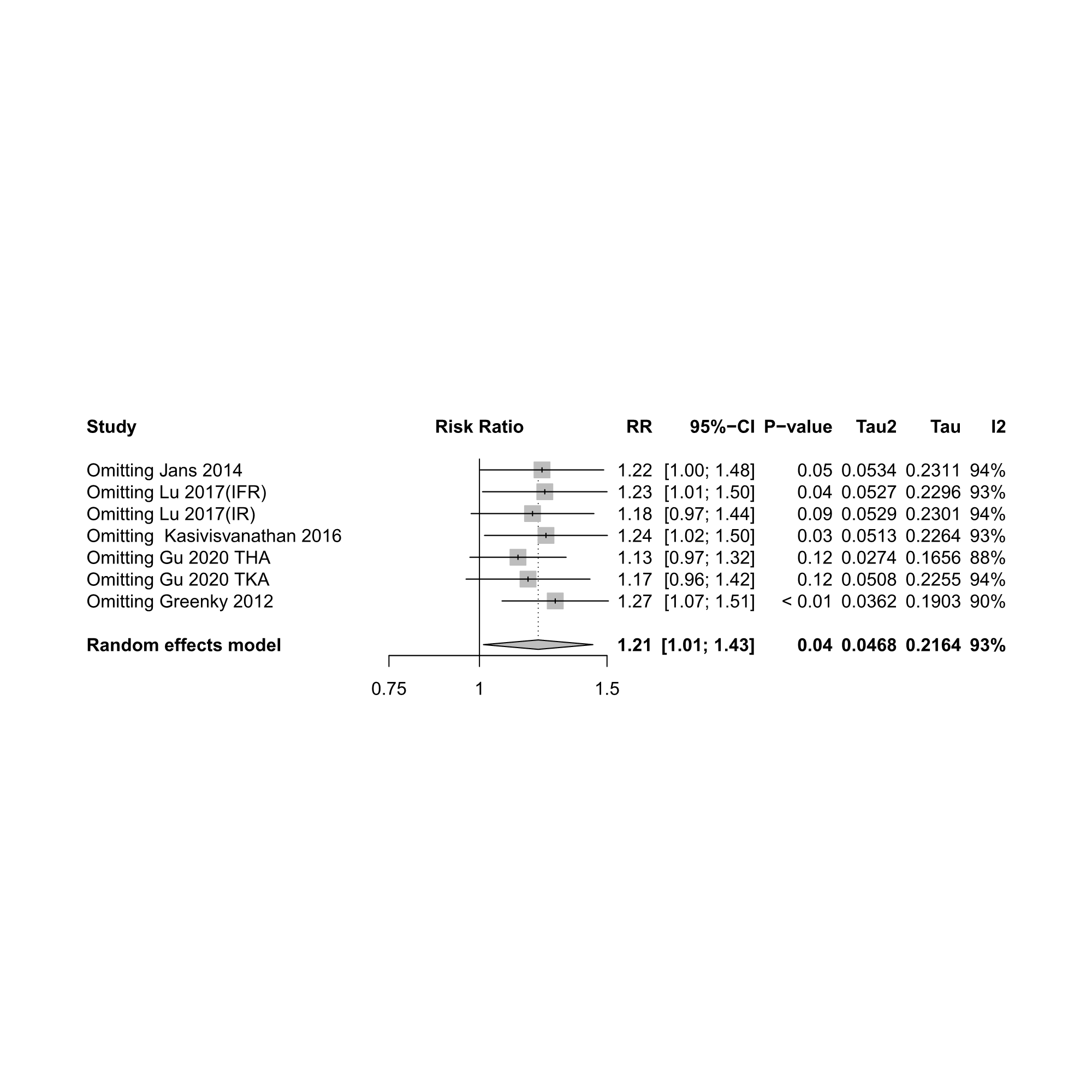 |
| --- |
| Fig. 3 |

1. Post-operative blood transfusion rate

As the heterogeneity was too large (I2 = 100%), we performed a sensitivity analysis and found that the heterogeneity did not show no significant change, indicating that the study was stable (Fig 4). This was confirmed by Egger's test (P = 0.4405), indicating that there was no publication bias.

| 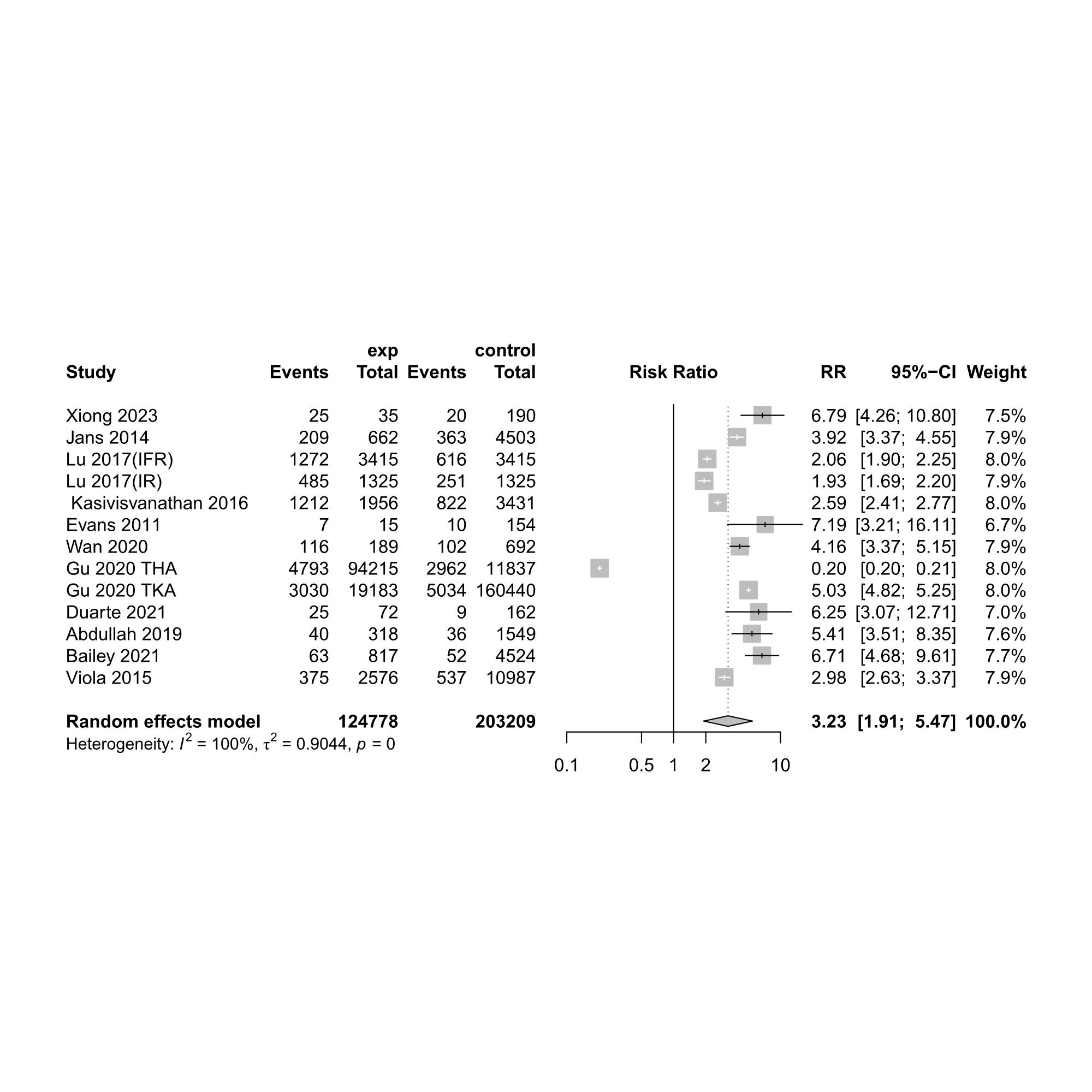 |
| --- |
| Fig 4 |

(5)DVT

Sensitivity analyses were performed and found that when excluding the study by Gu2020 TKA et al.(17) , the heterogeneity was drastically scaled down (I2=71%, RR=1.09 ,95%CI:[0.76, 1.56], P = 0.0001) (Fig. 5 ); however, this result should be viewed carefully because of its heterogeneity.

| 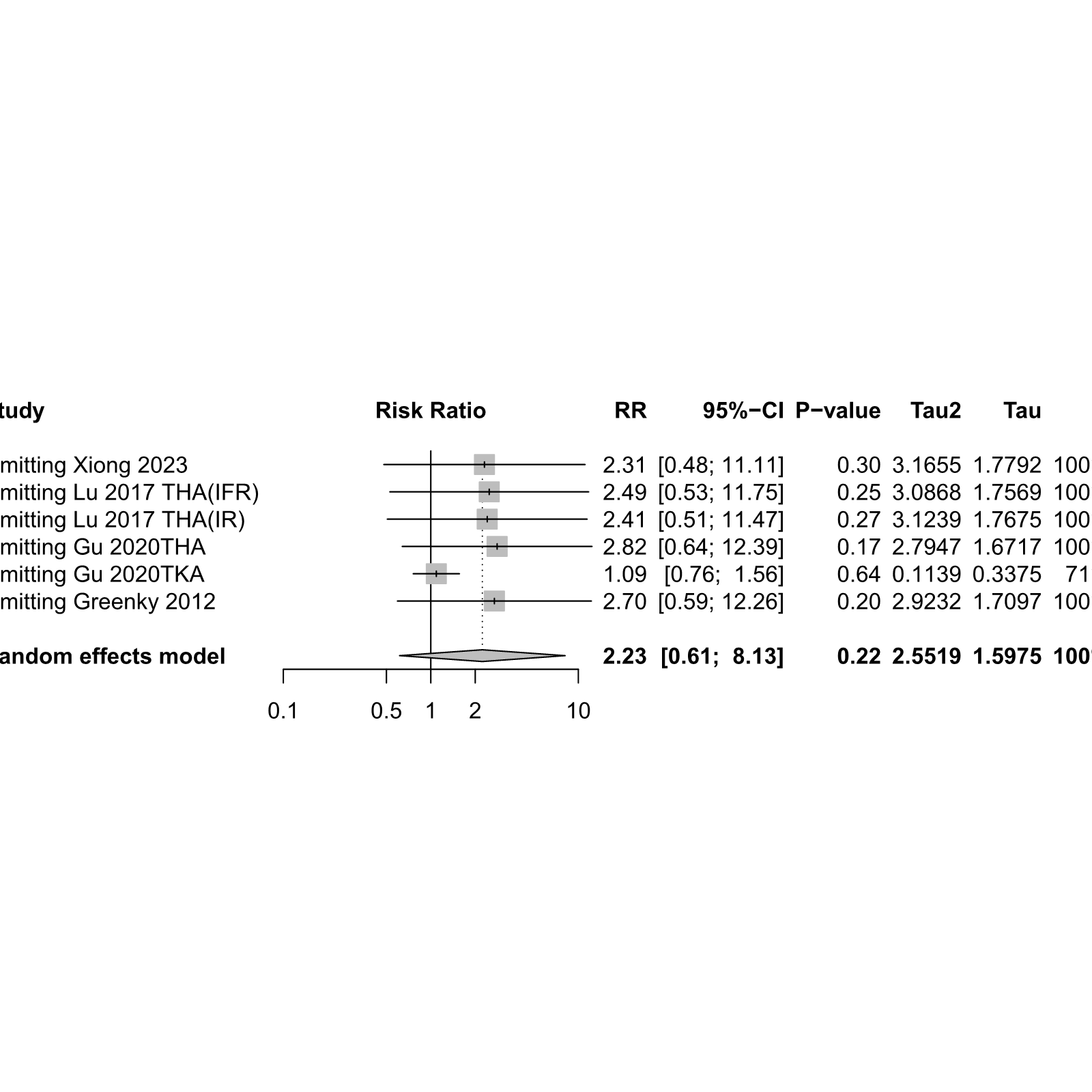 |
| --- |
| Fig. 5 |

1. Number of days in hospital

Because of the large heterogeneity, sensitivity analyses were performed and no significant change in the heterogeneity of the excluded studies was found on a study-by-study basis, suggesting that the included studies were relatively stable (Fig. 6). A funnel plot was drawn based on the results of days of hospitalization to assess publication bias. The graph was largely symmetrical (Fig.7), and we also performed an Egger test to confirm (P = 0.831) that there was no publication bias. This indicated that the risk of publication bias was low in this study.

| 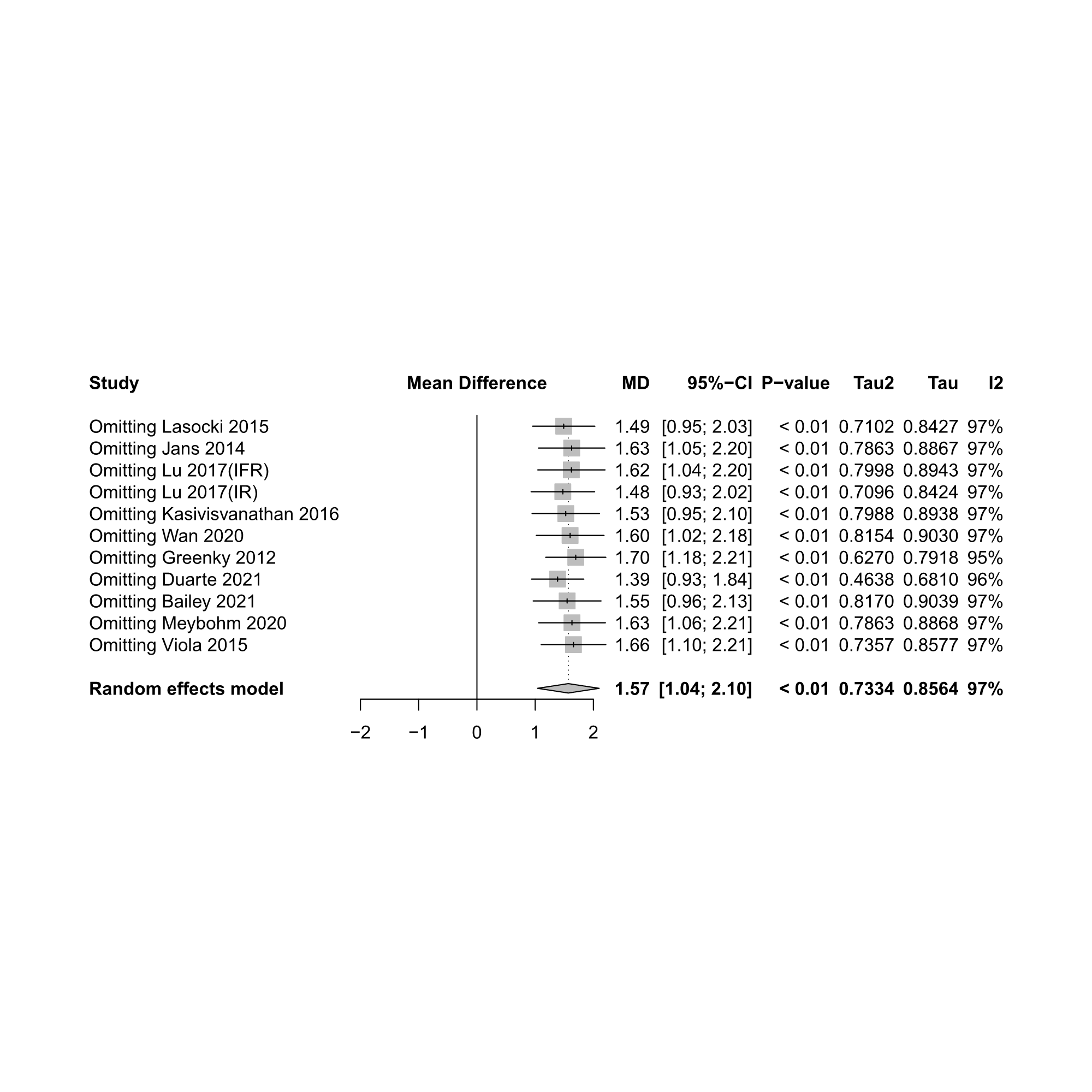 |
| --- |
| Fig. 6 |
| 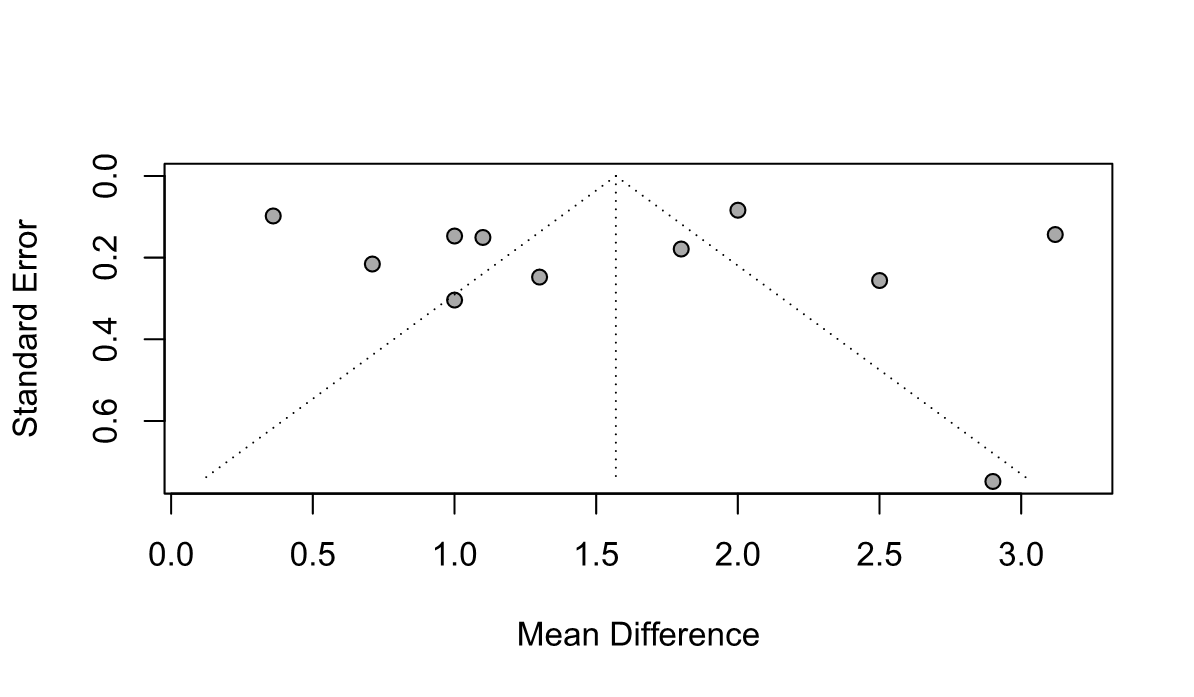 |
| Fig. 7 |

1. Rehospitalisation rate within three months

Because of the large heterogeneity, a sensitivity analysis was performed, and found that excluding this study of Gu2020 TKA(17) heterogeneity decreased slightly after (I2 =95%), but there was a large reduction in the risk of hospitalization (RR=1.70 ,95%CI:[1.19 , 2.44], P <0.01) (Fig 8 ).

| 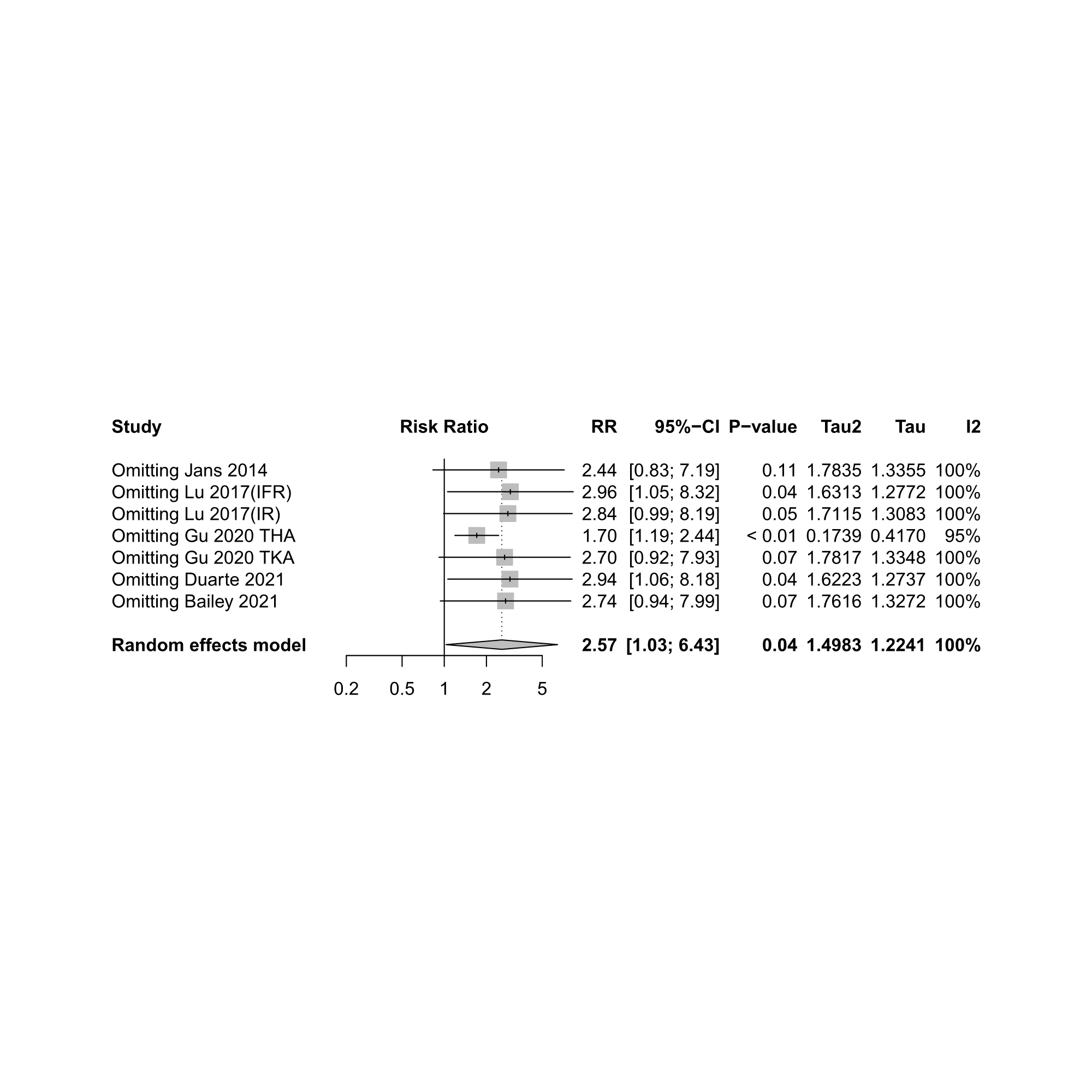 |
| --- |
| Fig. 8 |

1. Mortality

As heterogeneity was evident, a sensitivity analysis was performed and found that when this study, Greenky2012, was excluded(28) heterogeneity became 0, but the risk of mortality increased (RR = 4.69 ,95% CI:[3.79, 5.82], P < 0.01) (Fig 9).

| 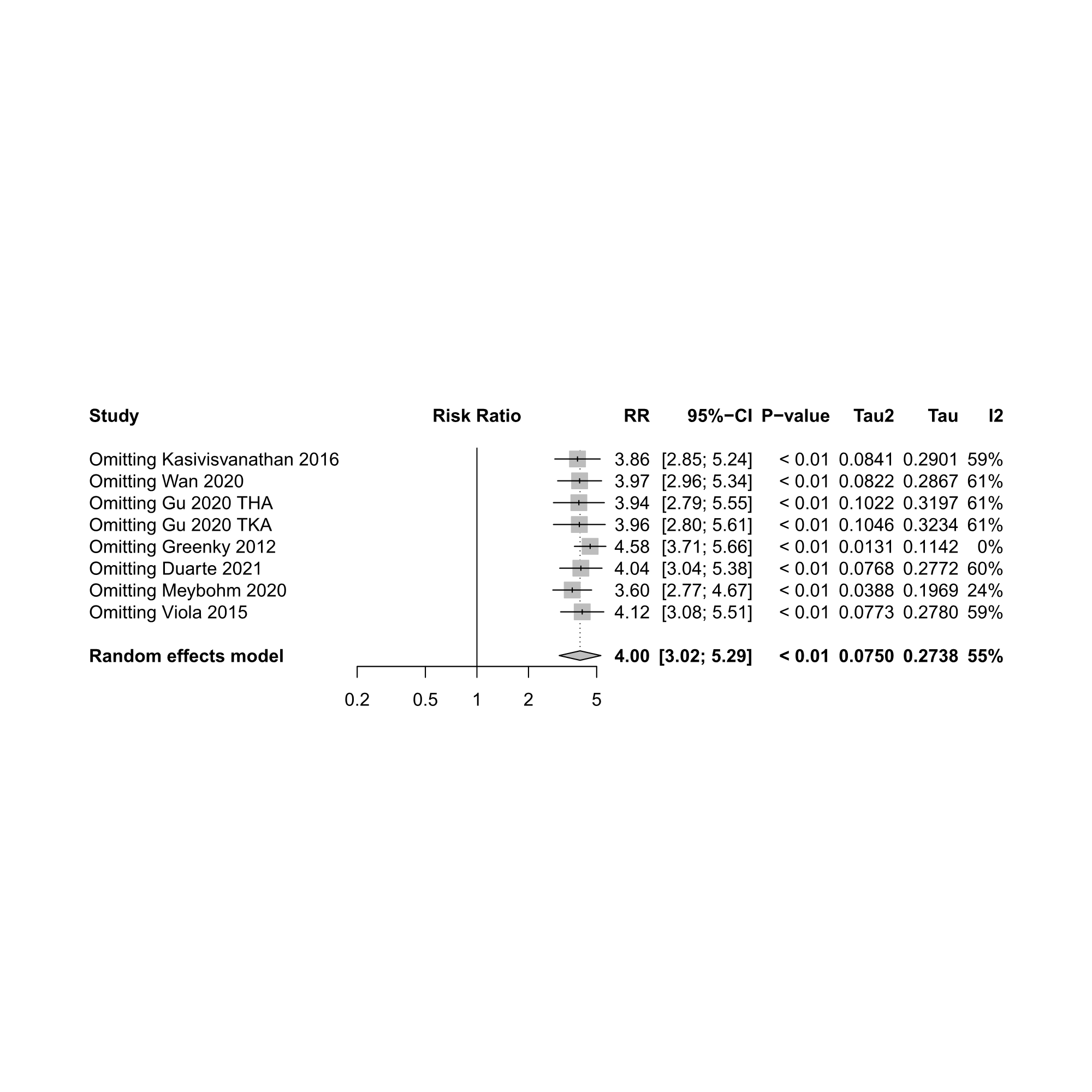 |
| --- |
| Fig. 9 |
